# Efficacy of quabodepistat alone and in combination with delamanid and bedaquiline against *Mycobacterium tuberculosis* in an NHP model evaluated by [^18^F] FDG PET/CT and bacterial burden

**DOI:** 10.1128/aac.00280-26

**Published:** 2026-09-16

**Authors:** L. E. Via, J. Sarathy, D. M. Weiner, T. Greenstein, M. Zimmerman, A. Abdi, J. D. Fleegle, F. Gomez, F. Kaya, M. K. Piazza, A. Vatthauer, B. Y. Sloan, K. M. Repoli, A. M. Walker, M. J. Woodcock, O. Egbelowo, K. Gausi, H. I. M. Boshoff, Y. Liu, D. Chadalavada, B. B. Aldridge, V. Dartois, C. E. Barry

**Affiliations:** 1 Tuberculosis Research Section, Laboratory of Clinical Immunology and Microbiology, National Institute of Allergy, and Infectious Diseases (NIAID), National Institutes of Health (NIH) 573242, Bethesda, Maryland, USA; 2 Tuberculosis Imaging Program, Division of Intramural Research, NIAID, NIH 469049, Bethesda, Maryland, USA; 3 Center for Discovery and Innovation, Hackensack Meridian Health 3139, Nutley, New Jersey, USA; 4 Department of Medical Sciences, Hackensack Meridian School of Medicine, Nutley, New Jersey, USA; 5 Department of Molecular Biology and Microbiology, Tufts University School of Medicine 12261, Boston, Massachusetts, USA; 6 Department of Biomedical Engineering, Tufts University School of Engineering 12261 , Medford, Massachusetts, USA; 7 Graduate School of Biomedical Sciences, Tufts University School of Medicine 12261 , Boston, Massachusetts, USA; 8 Division of Clinical Pharmacology, Department of Medicine, University of Cape Town, Cape Town, South Africa; 9 Otsuka Pharmaceutical Development & Commercialization Inc 155228, Rockville, Maryland, USA; 10 Comparitive Medicine Branch, Division of Intramural Research, NIAID, NIH 469049, Bethesda, Maryland, USA; 11 Department of Medicine, Centre for Infectious Diseases Research in Africa, Institute of Infectious Disease and Molecular Medicine, University of Cape Town, Observatory, Republic of South Africa; Queen Mary University of London, London, United Kingdom

**Keywords:** *Mycobacterium tuberculosis*, common marmoset, PET/CT, cavity, granuloma, non-human primate, antibiotic, quabodepistat, OPC-167832

## Abstract

Multidrug-resistant tuberculosis (MDR-TB) is challenging. Bedaquiline (B), delamanid (D), and pretomanid (P) are used in combination regimens with other anti-TB drugs for MDR-TB; however, agents with novel mechanisms of action are needed. DprE1 inhibitor quabodepistat (Q) was developed for combination use. In this study, Q alone or in combination with human-equivalent doses of D and/or B was evaluated in a marmoset TB model using *in vivo* imaging, with terminal bacterial counts from all lung lesions. Marmosets were infected with H37Rv *Mycobacterium tuberculosis* and allowed to develop the disease for 7 weeks. They were then administered a pre-treatment ^18^F-fluorodeoxyglucose positron emission tomography/computed tomography (18F-FDG PET/CT) scan and randomized into seven treatment groups (*n* = 5/group). At 2 weeks, the quabodepistat-treated groups showed a significant reduction in lesion volume compared to those without the agent in a post hoc analysis. After 2 months, the animals were euthanized, and the bacterial burden and histology were assessed. Marmosets treated with all agents showed reduced lung disease on PET/CT (>70%) compared to their pre-treatment disease volume. B reduced extrapulmonary bacteria more significantly than Q or D. Cavities and necrotic lesions from DBQ-treated animals had lower bacterial burdens than lesions treated with any of the single-agent or two-agent combinations. In a preliminary comparison, DBQ showed no statistically detectable difference from the standard-of-care regimen isoniazid-rifampicin-pyrazinamide-ethambutol (HRZE). Additionally, the activities of D and P monotherapies were similar in this model. The evaluation of these agents in the marmoset TB model supports further investigation of Q as a component of shorter TB regimens.

## INTRODUCTION

Tuberculosis (TB) has long been the most common cause of human mortality from infectious diseases, with the recent exception of the COVID-19 pandemic (1). Part of the difficulty with regimens designed to treat TB is the long duration of therapy and multiple side effects caused by the standard quadruple-drug therapy. New combination regimens often include older TB drugs (1, 2). As *Mycobacterium tuberculosis,* the causative agent of TB, has acquired resistance to drugs in the standard regimen, both access to effective alternative therapies and the side effects of second-line TB drugs have become costly challenges for healthcare systems in countries with high TB burdens (3).

Delamanid (D, Deltyba), bedaquiline (B, Sirturo), pretomanid (P), and linezolid have been approved for use in specific combinations in TB patients by the FDA, the European Medicines Agency, and/or other responsible regulatory agencies. B is a diarylquinoline that inhibits the c subunit of AtpE (encoded by Rv1305), reducing ATP pools in the bacterium and forcing a shift in energy metabolism to less productive pathways and a build-up of metabolites that eventually lead to bacterial cell death (4, 5). It is currently the pillar of multidrug-resistant (MDR) TB treatment, with the caveat that the development of resistance needs to be monitored (6). D, like P, is a prodrug that is metabolized to its unidentified active form by the *M. tuberculosis* F420 coenzyme and deazaflavin F420-dependent nitroreductase (7). It subsequently inhibits mycolic acid synthesis, with new evidence suggesting inhibition of the DprE2 subunit of decaprenylphosphoribose-2′-epimerase, thereby interrupting arabinose transfer into the arabinogalactan of the mycobacterial cell wall as well (8, 9). While these agents are fairly new, the question of which nitroimidazole should be promoted for widespread programmatic use is widely debated, as few studies have compared both agents head-to-head (10). Resistance to most of these agents has been detected during clinical studies and early programmatic implementation; therefore, the search for additional unique anti-TB agents continues (11–14).

The development of antimycobacterial drugs focuses on targeting novel pathways in *M. tuberculosis* that are distinct from those of current standard agents, and inhibitors of the steps in cell wall synthesis are commonly pursued (https://www.newtbdrugs.org/). The investigational agent quabodepistat (Q, OPC-167832) is one of several DprE1 inhibitors that disrupt different steps of arabinogalactan biosynthesis than the first-line agent ethambutol (15, 16). *In vitro*, Q is active against both drug-susceptible and MDR *M. tuberculosis* strains (15), and in several murine models, including C3HeB/FeJ mice (15, 17, 18). However, efficacy in murine models may not translate to clinical efficacy due to the differences in lesion development between humans and murine strains (19–21).

Tuberculosis lesions have been shown to exhibit heterogeneity in pathology, bacterial burden, and response to chemotherapy, even within the same individual (22–24). In both human TB and non-human primate (NHP) models, longitudinal ^18^F-fluorodeoxyglucose positron emission tomography/computed tomography (18F-FDG PET/CT) has become an important translational imaging biomarker by combining structural features of lung lesion burden by CT with metabolic measures of inflammatory activity reported by FDG uptake by PET. The imaging measures, including lesion volume, CT attenuation (Hounsfield units [HUs]), and FDG standard uptake values (SUV), correlate with disease severity and treatment response, but less directly with lesion bacterial burden (22, 25, 26). In human CT scans, regions with attenuation in the −500 to −101 HU or “soft” range were associated with tubercular nodules and tree-in-bud opacities by radiologists, whereas fibrosis, consolidations, and cavities were associated with high attenuations in the −100 to 200 HU or “hard” range. Reductions in FDG uptake and hard HU volumes have been shown to predict tuberculosis treatment success better than *M. tuberculosis* culture conversion at 2 months, and humans and NHPs demonstrate similar patterns of radiologic response (22, 25, 27). However, individual granulomas with similar imaging features can differ by several orders of magnitude in *M. tuberculosis* colony-forming units (CFU), indicating that longitudinal imaging and microbiological endpoints capture distinct but complementary aspects of disease progression and treatment response. Thus, combining radiologic and microbiologic measures may provide a more complete assessment of treatment response than either modality alone (22, 28).

Therefore, we conducted studies with Q in *M. tuberculosis*-infected common marmosets, as they develop cavitary disease, a pathology that puts patients at risk of relapse (26, 29)—and used PET/CT imaging of the lung for the longitudinal monitoring of the development of regions of high attenuation (or hard disease) volume as disease progression and pathological staging (27, 30). This approach enables visualization and quantification of infection dynamics in response to treatment. After observing no significant differences between D and P monotherapy in this model, we focused on evaluating Q, both alone and in combination with human-equivalent doses of D and B. Our goal was to determine the contribution of Q to bacterial clearance, particularly in organs where *M. tuberculosis* is often detected, as well as in radiologically and/or histologically defined pulmonary necrotic lesions and cavities.

## RESULTS

### Pharmacokinetics of bedaquiline, delamanid, and quabodepistat in marmosets

Naïve (uninfected) common marmosets were administered single doses of D, B, or Q individually, and plasma concentration–time profiles of the agents were determined. After several test exposures to the agents in various formulations, a dose that appeared to replicate the desired human exposure levels was tested individually in 21-day daily-dosing studies to determine each agent’s dose tolerability and steady-state pharmacokinetic profiles (Table 1). For D and Q, the once-daily doses selected in collaboration with Otsuka Pharmaceuticals were 10 mg/kg and 7.5 mg/kg, respectively. For P, a daily dose of 5 mg/kg was used as previously described (31). The increased metabolism of B in marmosets relative to humans necessitated the co-administration of 20 mg/kg 1-aminobenzotriazole (ABT), which reduced metabolic conversion but increased total B exposure more than we anticipated. The results of the initial 20 mg/kg dose of B in marmosets (Fig. S1A through D) suggest that B has further therapeutic potential, although at exposure levels that exceed those of FDA-approved dosing. With ABT, the B M2 metabolite reached about 200% of B exposure versus being only 25% of B exposure in humans (32, 33), making it necessary to include M2 in the modeling to select a human-equivalent dose since it would also contribute to bacterial killing (28). The B dosing scheme of 8 mg/kg (daily for 14 days) followed by 6 mg/kg (three times per week for 6 weeks) with 20 mg/kg ABT daily was selected for further study.

**TABLE 1:** Steady-state pharmacokinetic parameters of individual agents achieved in marmosets in comparison to clinical dosing strategies^*h*^

Drug	MIC (μg/mL)	Human		Marmoset				
		Dose (PO)	PK goal^*a*^ (µg · h/mL or µg/mL)	Dose (mg/kg)	AUC_(0–24 h)_ (µg · h/mL) range	*C*_max_ (µg/mL) range	*C*_avg_ (µg/mL) median	AUC/ MIC^*b*^
B^*c*^	0.06	400 mg x14 d, 200 mg 3×/wk	AUC 48; *C*_avg_:1.7	8 qd × 14 d 6 3×/wk	24–59	1.4–3.3	1.7	>400
(M2)^*d*^	0.3				58–113	5.6–2.2	2.8	>190
D	0.01	100 mg bid	AUC 3–8	10 qd	2–7	0.1–0.5	0.15	362
Q^*e*^	0.002^*f*^	30 mg qd^*g*^	AUC 2–3	7.5 qd	3–17	0.3–1.0	0.28 *C*_min_ 0.12 ± 0.7	>180

^*a*^ References: B (32, 34–36), D (37) and Q (17, 38).

^*b*^ T/MIC > 24 h for all three agents.

^*c*^ Marmosets dosed with B after a daily dose of ABT 20 mg/kg; dosing was after feeding with fat supplementation.

^*d*^ (M2) desmethyl B metabolite (33).

^*e*^ Q alone (see Table 2 for DDI).

^*f*^ Alone, with BSA shift MIC 0.016.

^*g*^ Dose assumption (38, 39).

^*h*^ Three to five animals contributed blood samples at 5 and 7 weeks for each agent investigated.

**TABLE 2:** Average AUC of quabodepistat by dosing duration in combination^*a*^

	Quabodepistat AUC_(0–24 h)_ (μg · h/mL)			
Dose(s)	Q Alone	With D	With B + ABT	With B + ABT + D
1	2.9 ± 0.9			
7–14	3.7 ± 2.1		18.4 ± 6.4	53 ± 26
14–28	8.8 ± 6.2	9 ± 2.5	*10.3 ± 1.4*	*29 ± 6.7*
>28	8.7 ± 2.8	11.1 ± 4.6	*10.1 ± 1.5*	*24 ± 5.1*
B (8 mg/kg × 15 days/6 mg/kg 3× wk) was administered after 20 mg/kg ABT (30 min prior)				

^*a*^ Three to five animals contributed blood samples for each agent or combination investigated. Italics: 3×/week dosing of B and Q with daily ABT.

To determine whether the targeted exposure levels for each drug were achieved during treatment, plasma samples were collected from M. *tuberculosis*-infected marmosets after 2, 5, and 7 weeks of treatment with each agent at the estimated peak and trough time points for each agent. The 24-h exposures (area under the curve [AUC] values) measured for each drug individually at steady state are summarized in Table 1 along with the published clinical pharmacokinetic (PK) parameters for reference. The bedaquiline dosing scheme achieved the desired human-equivalent exposure and average plasma concentration (Table 1) (28, 32, 34). The combined exposure to B and its five times less active M2 metabolite gave an AUC/MIC ratio of >900, which was close to the >700–800 combined AUC/MIC ratio for the 400 mg (daily for 14 days) followed by 200 mg (three times per week) used in successful multidrug-resistant tuberculosis treatment (40).

No adverse clinical reactions were observed in the 2-month marmoset efficacy study. Minimal drug–drug interactions were observed before 1 month of treatment, although ABT was found to increase Q’s exposure over 14 days in naïve marmosets (Fig. S2). However, during the second month of therapeutic monitoring, Q showed an increased AUC alone when administered with B + ABT, or with D, and had significantly increased exposure when administered with both D and B, which is at least partially due to concomitant dosing with ABT (Table 2 and Fig. S2). Conversely, the AUC of D did not significantly change when combined with either Q or B, despite consistent co-administration of B and ABT (Table S1). D plasma exposure levels were within the range of those in patients with TB receiving 100 mg twice daily (37).

### Treatment of infected marmosets reduces tuberculosis lung disease

Without treatment, marmosets experience progressive disease with increasing lung disease volume, FDG uptake, and bacterial burden accompanied by weight loss and dehydration until the animals become moribund around 8–10 weeks post-infection (PI) (27). If effective treatment is begun at 6–8 weeks PI, disease progression can be reversed (27, 30). In this study, marmosets were infected with *M. tuberculosis* by aerosol exposure, and the disease was allowed to develop for approximately 7 weeks. FDG PET/CT scans of the marmosets were collected immediately before treatment initiation and every 2 weeks thereafter to monitor the treatment response. Data from the marmosets included in this analysis represent a subset of the animal treatment groups that were analyzed in two other published studies using different analytical methods and foci (28, 31).

We examined our earlier single-agent treatment data to determine whether there was an advantage of one nitroimidazole over the other in our model. A group of marmosets was randomized to receive human-like exposure to P (5 mg/kg) or D (10 mg/kg) daily for 2 months, which promoted the survival of the marmosets to the end of the 15-week study period, improved radiologic measures of pulmonary disease, and reduced the number of lung and non-pulmonary organ samples that were *M. tuberculosis* culture-positive compared to untreated marmosets. Bacterial load reduction was not significantly different between P and D in the lungs, extrapulmonary organs, or cavitary lesions (Fig. S3A through G). D was selected for the subsequent combination regimen assessments as clinical studies of D + Q combinations were being planned at the time (41).

**Fig 1:**
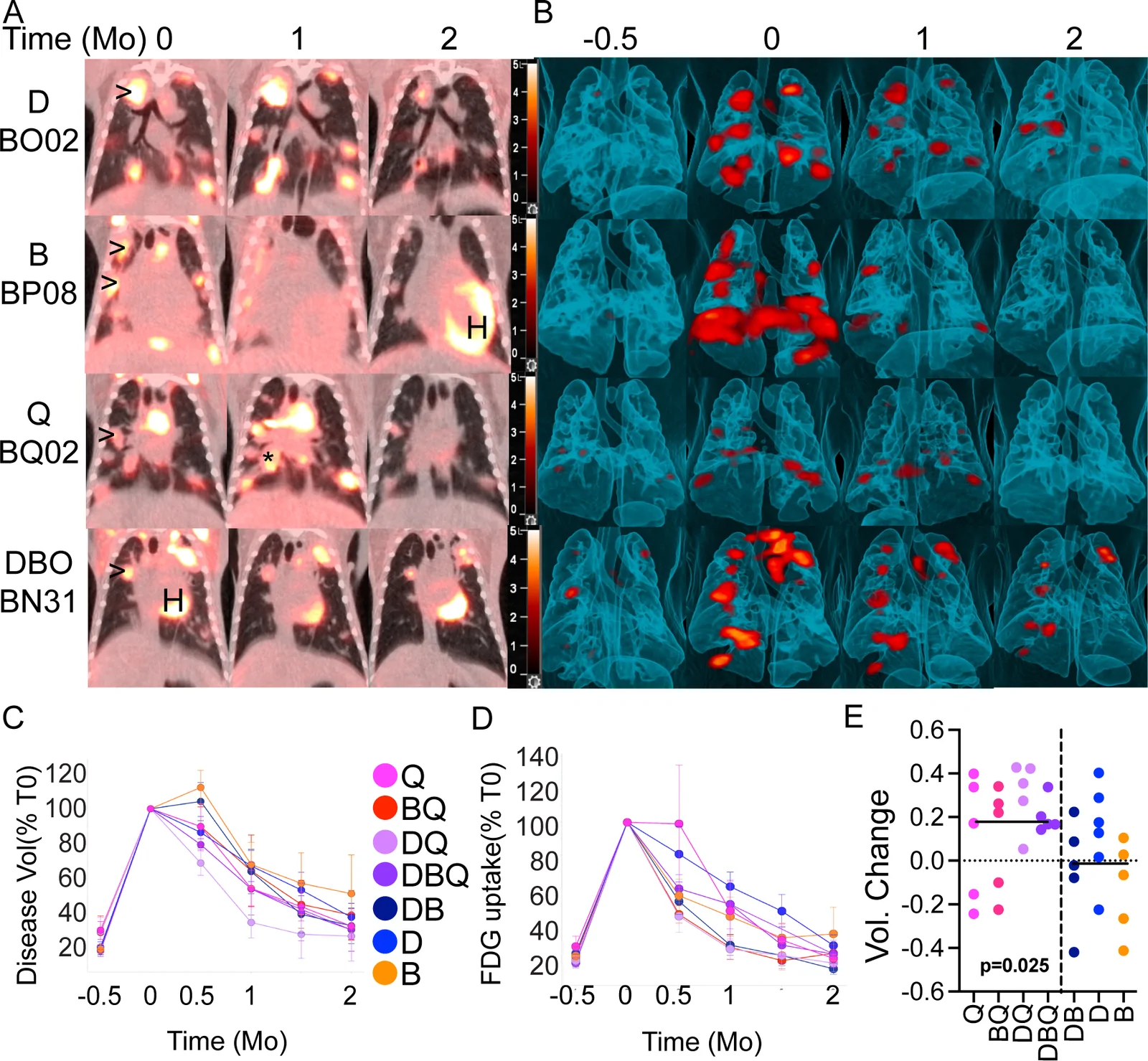
Response to anti-TB therapy as revealed by coronal PET/CT images and 3D maximum image projections in representative marmosets. Treatments and animal ID are indicated on the left of each row of images. (**A**) Coronal FDG PET/CT images show several individual lesions (arrowheads) and a cavity (*) that shrink and become less FDG-avid in the serial images quantified in panels C and D. The SUV activity scale applies to panel A. An H marks FDG uptake by the heart that is unrelated to *M. tuberculosis* infection and is excluded from the maximum intensity projections (MIPs). (**B**) MIPs of the relative FDG uptake in the marmoset lungs from 2 weeks before treatment start (−0.5), pre-treatment (0 month [mo]), and during treatment (1 mo and 2 mo). The 3D MIPs of the lungs show the CT structures (black to light blue), with higher-density tissue as lighter blue. Many lesions appear as light spheres or cup-shaped structures in the less dense, darker lung tissue. The metabolic activity of the lesions is indicated in relative scale from no color (low), red (medium), to yellow (high) FDG uptake in lesions. FDG uptake increased from −0.5 to 0 (treatment start), then decreased during the 1 month after treatment start, and little activity remained after 2 months of treatment as quantified in panel **D**. (**C**) Treatment initiated immediately after time point 2 (TP2), and most treatments reduced both mean hard disease volume and (**D**) mean FDG uptake of the pulmonary lesions (both normalized to TP2), although there was not a significant difference among the treatments at that time point (**C and D**). Error bars in panels **C and D** represent the standard deviation of the means. (**E**) In a post hoc exploratory analysis, the proportional change in total lesion hard volume after 2 weeks of treatment was significantly higher in regimens that included quabodepistat (to the left of the dashed vertical line, 0.177 mean change) versus those treatment groups without Q to the right (−0.001 mean change) (*P* = 0.025, *t*-test). See Fig. S5 for a confirmatory analysis. In the graph, each dot represents the change in total disease volume in a marmoset, and the lines indicate the means of the proportional change. The color assigned for each treatment remains the same throughout (**C–E**). A recently published, lesion-centric analysis of this data found that severe lesions specifically showed early reductions in hard volume during treatment with the combination of BQ (28).

In addition to treating groups of marmosets with single drugs B, D, or Q, groups were administered one of the three two-drug combinations (BD, BQ, or DQ) or the three-drug combination DBQ daily for 2 months (*n* = 7 groups). Lung lesions were identified by comparison with a pre-infection CT scan and captured into individual volumetric regions of interest (VOIs) (Fig. 1A; Fig. S4). The VOIs for each lesion were tracked throughout the treatment. Features such as hard (−100 HU to 200 HU) and soft (−500 HU to −101 HU) CT attenuation volumes, as well as other CT and PET features, were recorded (25, 30). The volumes of each marmoset’s lesions were combined to obtain the total volume of lung lesions per animal at each time point (e.g., Fig. S4B), which was averaged for each treatment group (e.g., Fig. S4C). A similar process was followed to calculate FDG uptake in the lesions (Fig. S4D through F). The distribution of marmoset total lesion volume at the start of treatment was similar in each group (Fig. S4G). Over time, treatment reduced the disease volume (Fig. 1A and C) and FDG uptake of lung lesions in all marmoset groups (Fig. 1B and D). The rate of initial treatment response after 2 weeks (or 0.5 months) differed among the treatment groups. To visualize this, lesion volume and FDG uptake for each animal were normalized to the pre-treatment measurement, and the mean percent change was plotted (Fig. 1C and D), showing a trend toward differences in disease volume change among treatment groups (ANOVA, *P* = 0.07). We then examined the disease volume change in all animals dosed with Q in comparison to animals that did not receive Q in a post hoc exploratory analysis, and we found that animals administered Q-containing regimens had significantly greater reductions in disease volume (*t*-test, *P* = 0.025; Fig. 1E and Fig. S5), indicating an association between Q-containing regimens and greater reduction in high-attenuation lung volume reduction early in treatment that B- and D-containing regimens did not demonstrate (Fig. S5). Similarly, in Greenstein et al., unsupervised clustering of individual lesions from marmosets treated with BQ revealed that Q, when combined with B, reduced PET/CT-measurable disease pathology more rapidly than other treatments in the first month of therapy (28). After 2 months of treatment, all seven groups of marmosets showed improvement in disease measures; the CT volume of disease showed a 73% median reduction (IQR 53%–85%, Fig. 1C) and the FDG uptake decreased by a median of 85% (IQR 73%–92%, Fig. 1D) from baseline values, but the decreases were not significantly different among the individual treatment groups.

### Bacteriologic responses to treatment

**Fig 2:**
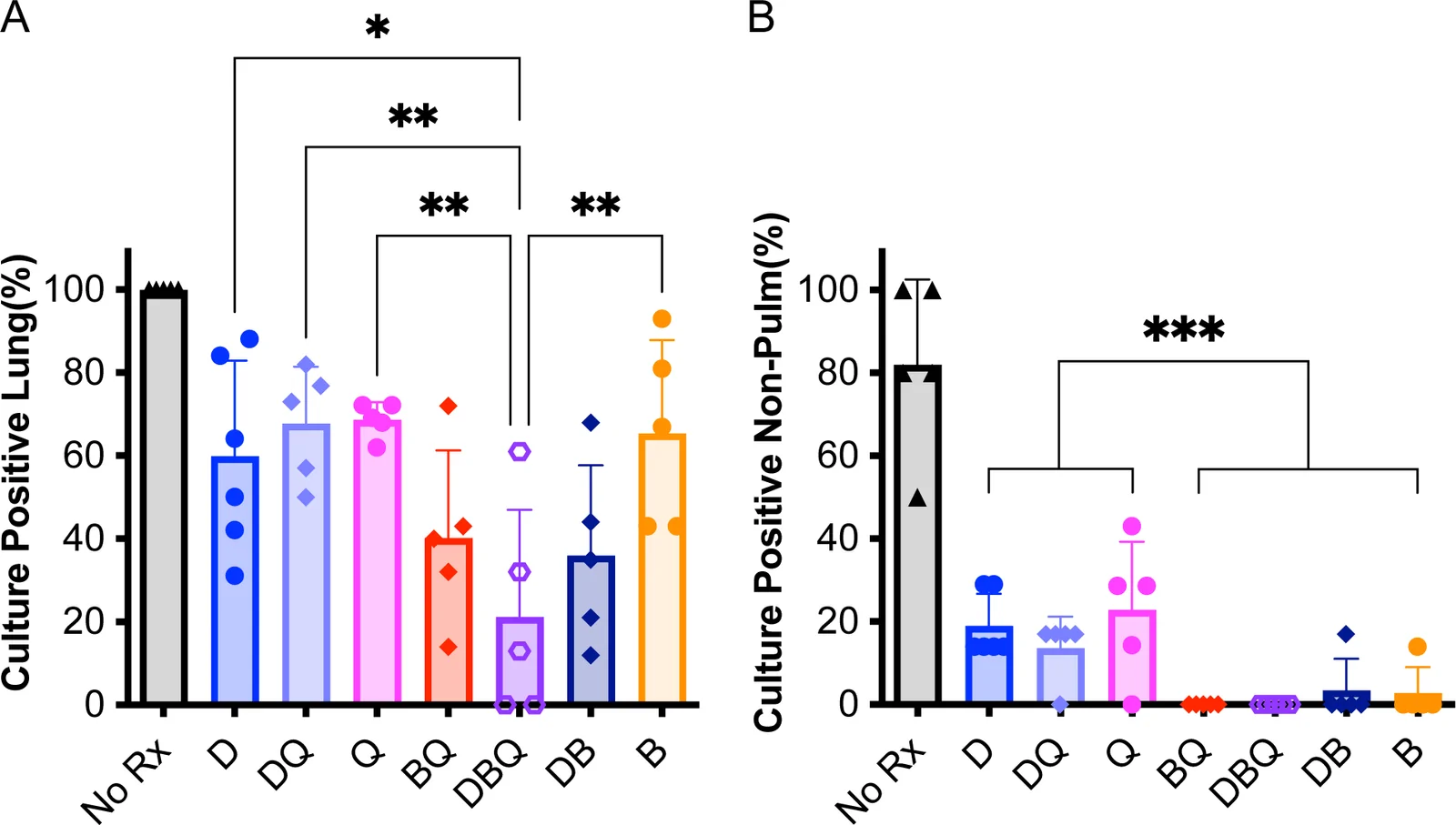
Culture positivity rate of lung lesions and extrapulmonary organ samples obtained after 2 months of treatment was reduced in marmosets treated with delamanid, bedaquiline, quabodepistat, and combinations. (**A**) The drug treatments all reduced the number of culture-positive lung samples compared to the no-treatment control (*P* < 0.001, ANOVA, HS-MCT, no line indicated). Among the single-agent treatments, delamanid, bedaquiline, and quabodepistat significantly reduced the proportion of culture-positive lung samples by 30%–40% compared to no treatment (*P* < =0.02, ANOVA, HS-MCT, not indicated) but were not different than each other. In addition, the combination of DBQ significantly reduced culture positivity more than any single agent and DQ (*P = 0.01, **P < 0.007, ANOVA HS-MCT) but was not significantly different than BQ or DB treatments. Similarly, two-agent treatments were not detected as statistically different from each other. See Fig. S6 for a component-level analysis of the regimens; each agent was associated with lower culture positivity, with bedaquiline associated with the largest estimated reduction in both tissue types. (**B**) Culture-positive extrapulmonary samples were significantly reduced in the treated groups compared to untreated marmosets (*P* < 0.001, ANOVA HS-MCT, not indicated). Interestingly, the animals treated with bedaquiline or any combination including bedaquiline were significantly more likely to have no positive extrapulmonary cultures than the groups without bedaquiline (*** *P* = <0.001; Fisher’s exact test).

Unlike rhesus macaques (23) or New Zealand White rabbits (42–44), self-sterilization of TB lesions or prolonged subclinical disease is rarely observed in *M. tuberculosis*-infected marmosets in the absence of drug treatment, unless the animals receive an extremely low infection inoculum (45). Hence, we first compared the proportion of lung samples without detectable bacteria among the treatment groups after 2 months of treatment to gauge drug efficacy (Fig. 2A). In addition to sampling all the observed lung lesions, three visibly normal lung specimens were collected from each marmoset and plated on M7H11 agar for bacterial enumeration. All seven treatment groups had fewer culture-positive lung samples than untreated marmosets sampled between 7 and 10 weeks post-infection (Fig. 2A; *P* < 0.001, ANOVA with Holm-Sidak’s multiple comparison test [HS-MCT]). D, B, and Q individually decreased the rate of culture positivity by 30%–40% compared to those marmosets without treatment. In addition, DBQ-treated animals had significantly fewer positive lung samples than the single-drug and DQ combination-treated animals (*P* ≤ 0.01, ANOVA with HS-MCT). The differences between the BQ and DB combination treatment groups were not statistically significant (Fig. 2A). Additionally, each of the component agents B, D, and Q was associated with a lower proportion of culture-positive lung tissue samples in a post hoc factorial linear regression analysis (Fig. S6). We also assessed the liver, spleen, right kidney, subcarinal, hilar, and axillary lymph nodes, which have been shown to contain *M. tuberculosis* in previous studies (27), to measure the extrapulmonary bacterial burden. As with the lungs, all treatments significantly reduced the number of bacterial CFU when compared with untreated infected animals (Fig. 2B; Fig. S6E). Interestingly, marmosets treated with a B-containing combination had significantly fewer culture-positive lymph node and non-pulmonary organ samples than those treated with other combinations (*P* < 0.0001, Fisher’s exact test), suggesting that B could be especially efficacious in well-perfused extrapulmonary tissues without exposure barriers.

### Lesion-specific responses to treatment with the DBQ regimen

In humans, individuals with cavitary lesions are more likely to relapse than those without radiographic evidence of cavities (29, 46), and we observed that cavitary lesions were more difficult to sterilize in marmosets (27). Multiple lines of published evidence suggest that the drug tolerance of non-replicating persistent bacteria in the caseous core of TB cavities and necrotic lesions directly influences treatment outcomes (47, 48). Therefore, we examined the concentration-kill profiles of D, B, and Q in *ex vivo* rabbit caseum using previously reported methods (49) (Fig. S7). MBC_90_ was defined as the minimum drug concentration required to kill 90% of resident *M. tuberculosis* in caseum homogenate. B demonstrated potency against caseum *M. tuberculosis ex vivo* (MBC_90_ of 4.7 µM) (Fig. S7A). D was unstable in caseum, as it was in plasma, owing to rapid metabolism by albumin (50), preventing an MBC_90_ determination using the standard protocol. To address this, we implemented a daily-replenishment protocol for D only in the *ex vivo* caseum assay, to more accurately reflect the potency of D against these nonreplicating persisters in patients receiving daily doses. Caseum homogenate, prepared using standard methods, was exposed to delamanid at the standard drug concentration range of 0.03–128 µM on day 0, followed by daily additions of the drug for 6 days, prior to sampling for CFU plating on day 7. Average D exposure at each incubation level over the 7-day assay was tracked daily by liquid chromatography-tandem mass spectrometry. Insignificant changes in incubation volume and DMSO content resulting from the replenishment did not affect CFU burden determination. Using this protocol, we determined that D could kill *M. tuberculosis* in *ex vivo* caseum (MBC_90_ of 7 µM daily) (Fig. S7B), as is the case for pretomanid (49). Q displayed limited bactericidal activity against non-replicating bacterial populations (MBC_90_ >512 µM) (Fig. S7C), similar to other DprE1 inhibitors (17, 51) and other inhibitors of cell wall biosynthesis pathways in this *ex vivo* caseum model (49).

**Fig 3:**
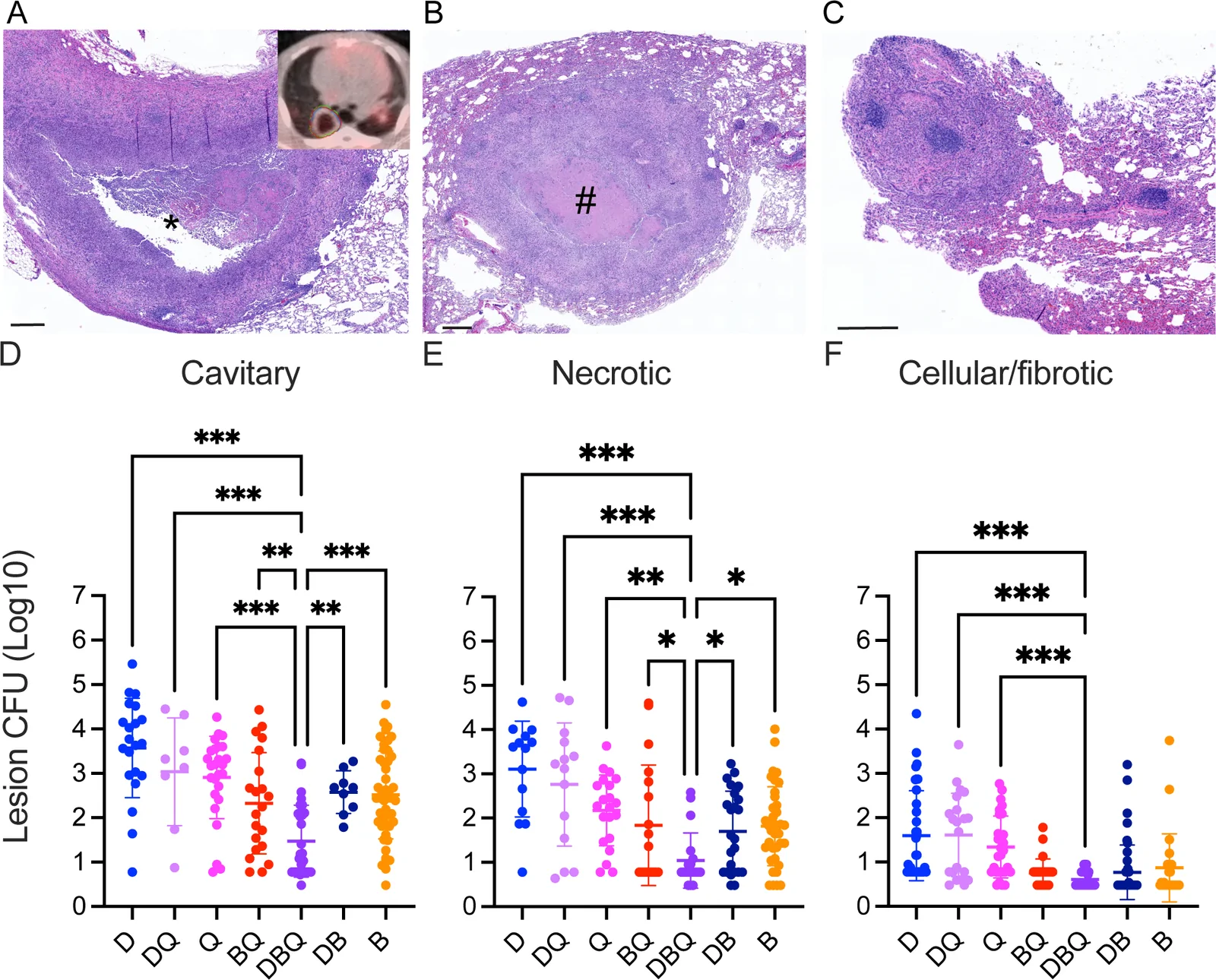
DBQ combination treatment significantly reduced the bacterial loads in cavities and necrotic lesions compared with the individual agents and two-agent combinations. Lesions were identified as (**A**) cavitary, (**B**) necrotic, or (**C**) cellular/fibrotic lesions by radiological images, gross pathology, and H&E histopathology. (**A**) Many cavities observed had residual necrotic material, as shown. The inset is a PET/CT image of the same 5 mm cavity. (**A–C**) Scale bars, 400 µm; * cavity lumen with residual debris; # region of necrosis. (**D and E**) Comparing the bacterial burdens of the individually identified lesions, the DBQ treatment group had significantly lower bacterial loads in cavity and necrotic lesions compared to the other treatment combinations (*P* < 0.033 [*], <0.002 [**], <0.001 [***], ANOVA, HS-MCT). (**F**) In cellular/fibrotic lesions, the DBQ group had significantly lower bacterial loads than the groups that did not contain B (*P* < 0.001 [***], ANOVA, HS-MCT), but not those with B. Each dot represents an individual lesion tracked and collected. The number of lesions and the animals contributing are summarized in Table S2. The results of the sensitivity analysis using a linear mixed-effects model with animal-level clustering are shown in Table S3. See Fig. S7 for single agent to untreated lesion comparisons. Among the two-agent groups, BQ and BD both reduced bacterial CFU significantly more than DQ in the cavitary (**A**) and cellular lesions (**C**) (*P* < 0.001, ANOVA, HS-MCT). In necrotic lesions (**B**), DB was superior to DQ (*P* = 0.03, not indicated on graphs).

Because individual tuberculosis granulomas are known to exhibit substantial heterogeneity in bacterial burden and treatment response, lesions were analyzed as the biologically relevant unit, consistent with prior nonhuman primate, rabbit, and human tuberculosis studies (23, 24, 43, 52, 53). Because cavities have been shown to be difficult to sterilize, we sought to determine whether the DBQ regimen reduced bacterial burden within cavitary lesions relative to comparator regimens; therefore, we examined the performance of Q and Q-containing regimens in specific lesion types: cavities, necrotic lesions, and cellular/fibrotic lesions lacking necrotic centers. PET/CT-identified lesions were grouped into three major categories at necropsy. Categorization was confirmed by histopathology using hematoxylin and eosin and/or Masson’s trichrome staining (e.g., Fig. 3A through C). The logarithmic function of bacterial burden, enumerated as CFU, of the lesions in marmosets treated with a single agent was first compared to those of untreated animals (Fig. S7D through F). We observed that Q and B were more active than D in necrotic and cavitary lesions. Considering the relatively poor bactericidal activity of Q against non-replicators in caseum (Fig. S7C), Q’s efficacy in marmosets could be attributed to its high potency against replicating bacterial populations in the host, in addition to its good distribution in lesion compartments (17). When comparing among treatment groups, DBQ was more active than all single-agent and two-agent combination treatment arms in cavities and necrotic lesions (Fig. 3D and E; *P* ≤ 0.03 ANOVA with HS-MCT) but not fibrotic lesions (Fig. 3F), suggesting that all three agents contributed to the collective efficacy of DBQ in lesions with necrosis. To assess the impact of within-animal correlation, we conducted a sensitivity analysis using a linear mixed-effects model with animal as a random intercept. Intraclass correlation coefficients (ICCs) indicated low clustering for cavity lesions (ICC = 0.177) and moderate clustering for necrotic (ICC = 0.226) and cellular fibrotic lesions (ICC = 0.220). The mixed-effects analysis yielded effect estimates that were consistent with the primary lesion-level analyses but produced a more conservative statistical effect size by accounting for within-animal clustering (Table S3). Consequently, some pairwise comparisons no longer reached statistical significance after adjustment, although the overall pattern of treatment effects remained similar. DB and BQ showed significantly greater activity than DQ in some lesion types (*P* < 0.001, ANOVA, HS-MCT), indicating that B is a critical driver of lesion sterilization in combination regimens. Because Q accumulated more than anticipated, and especially when combined with the full DBQ regimen, we evaluated whether there was a significant exposure–response relationship between the Q AUC_(0–24 h)_ and the microbiologic outcomes, including lung Log_(10)_ CFU. Pooling the data from the Q, BQ, DQ, and BDQ groups in a multivariable model accounting for regimen (Fig. S8A), we found that Q AUC_(0–₂₄ h)_ was not significantly associated with total lung CFU (*P* > 0.8), mean lesion CFU (*P* = 0.45), median lesion CFU (*P* = 0.46), or log-transformed total CFU (*P* = 0.65), indicating a lack of within-regimen exposure–response relationship for these endpoints (Table S4). Overall, these findings suggest that, within the exposure range achieved in this study, Q alone, or in a specific combination, did not exhibit an exposure-dependent effect on CFU burden. A similar analysis was conducted for D and B + M2 (Fig. S8B through D and Table S4) and B alone (not shown), and there was no significant association in the multivariable linear regression. Interestingly, there was an inverse association observed in the D alone and B + D groups with remaining Log_10_ CFU, whereas the D + Q group showed a positive association, and the DBQ group showed no association (Fig. S8B).

### The DBQ regimen was not detectably different from the standard-of-care regimen in *M. tuberculosis*-infected marmosets

**Fig 4:**
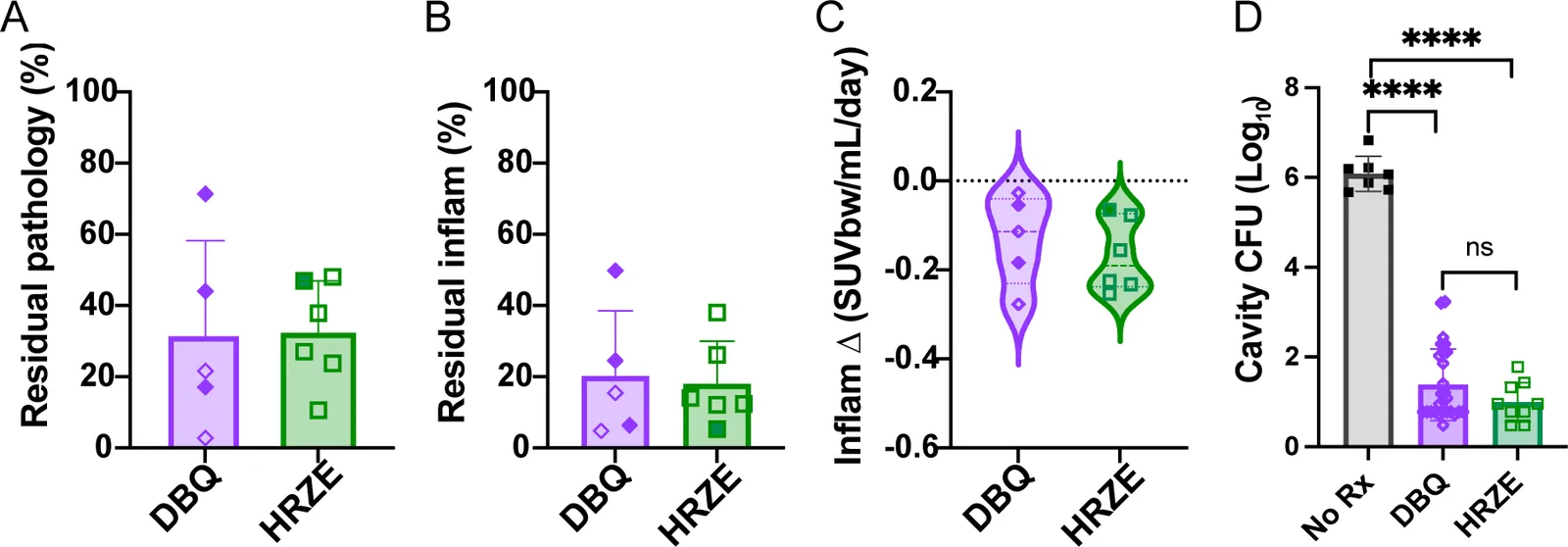
The DBQ combination treatment was not significantly different than HRZE in the marmoset model of progressive tubercular disease. We compared the outcomes of marmosets treated with DBQ with those of marmosets treated with HRZE for 8 weeks. The results indicate (**A**) residual pathology percentage measured in the 2-month CT scan and (**B**) residual inflammation percentage measured from the FDG PET scans were not different. In addition, (**C**) there was no significant difference in the rate of reduction of FDG uptake over the 2-month treatment. The dashed line in the violin plot shows the median and the dotted lines the quartiles of the data. (**D**) Both regimens reduced cavitary CFU in the lesions (****, *P* < 0.0001 ANOVA, HS-MCT), but there was no significant difference in the two regimens’ performance (*P* = 0.083). A sensitivity analysis using a linear mixed-effects model with animal as a random effect yielded the same conclusion as the ANOVA (ICC = 0.22, *P* = 0.56). See also the measured remaining volume of pathology and total remaining FDG uptake in these animals in Fig. S9.

Given that the DBQ combination significantly reduced lung CFU and had very low culture-positive sample rates in extrapulmonary tissue, we compared 2 months of DBQ treatment with data for 2 months of HRZE treatment reported previously (28, 31). The residual pathology and inflammation at the end of the treatment were not statistically detectably different (Fig. 4A and B; Fig. S9A and B), nor were the rates of change of the disease volume or FDG uptake at 2 months of treatment (Fig. 4C; Fig. S9C). In addition, when comparing the remaining bacterial load in the total lung or mean cavities or in individual cavitary lesions, the difference between the two regimens was insignificant in this preliminary comparison (Fig. 4D; Fig. S9D and E).

## DISCUSSION

In this study, quabodepistat monotherapy reduced bacterial burden in cavitary lesions relative to untreated controls, and the lower cavitary-lesion burden observed with DBQ than with DB in the primary lesion-level analysis was consistent with an additional contribution from quabodepistat. These results support its continued investigation as a new component of TB treatment regimens. These findings are important because although higher doses of rifampicin and rifapentine have shown promise for increasing cure rates and shortening treatment, treatment challenges remain (54–56). Additional anti-TB agents with novel mechanisms of action are still needed to combat increasing *M. tuberculosis* drug resistance, the complexity of TB treatment in people with HIV co-infection, and the emergence of resistance-associated variants conferring cross-resistance to newer agents such as bedaquiline (57–59). The targeting of DprE1 by quabodepistat is a mechanism of action not yet represented among the approved agents for tuberculosis treatment, although it is considered a highly vulnerable target among the genes involved in cell envelope biosynthesis (60). The quabodepistat-containing regimens were associated with greater early reduction in high-attenuation CT lesion volume in a post hoc exploratory analysis (Fig. 1 [28]). It will be interesting to determine whether other DprE1-inhibiting agents demonstrate similar associations.

Q is highly bactericidal in THP-1 intracellular assays and several murine models of *M. tuberculosis* infection, both as a single agent and in combination regimens. Furthermore, lesion-centric pharmacokinetic analysis revealed that Q partitions well into lesion compartments, producing favorable exposure levels throughout the dosing interval (15, 17, 18). In marmosets, plasma concentrations of Q exceeded the maximal intracellular bactericidal activity concentrations (>4 ng/mL) (15) and the serum-adjusted MIC by several folds, even at 24 h post-dose, suggesting that Q exposure was replete. In this study, we observed that Q was highly active in an NHP model of cavitary tuberculosis despite its limited bactericidal activity in *ex vivo* caseum. Interestingly, a previous study reported that Q was highly efficacious in the C3HeB/FeJ mouse model of TB infection, in which much of the pathogen population resides in necrotic type 1 lesions with caseous cores (17). Indeed, a clear differentiation in Q treatment response between C3HeB/FeJ mice that did and did not display pronounced Type I caseous lesions aligns with our observation of Q inactivity in the *ex vivo* caseum assay. Hence, Q’s overall efficacy in infected marmosets must be attributed to other intracellular and extracellular *M. tuberculosis* populations in the host (15). Q exposure levels at the ranges observed here appeared to be above the ascending effective dose range, as we did not observe an exposure-dependent effect on bacterial load (Fig. S8A and Table S4).

In this study, Q was clearly associated with reductions in high-attenuation lung volume early in treatment with Q-containing regimens (Fig. 1E); this is not the case for B or D (Fig. S5). On the other hand, when culture positivity was examined, Q, B, and D all demonstrated statistically significant contributions, with B having the highest (Fig. S6). This may indicate varying temporal contributions of individual drugs during treatment, perhaps depending on tissue penetration and mechanism of action. This reinforces the need for multiple drugs in the treatment of TB.

DBQ-treated cavities and necrotic lesions had lower bacterial burdens than any of the underlying single- or two-agent combination treatment arms, suggesting that all three agents contribute to the regimen’s activity. This contrasts with the bedaquiline–pretomanid–linezolid (BPaL) MDR-TB regimen, where linezolid was found to make an insignificant contribution to the *in vivo* efficacy of the three-drug regimen (28, 31, 61). Although B required more time to show disease volume reversal than the other two agents by CT, it was especially effective in clearing bacteria from the well-perfused extrapulmonary organs (Fig. 2), but this does not necessarily apply to TB meningitis or other forms of TB in compartments with restricted drug penetration (62, 63). TBAJ-876, a next-generation diarylquinoline (64) that reaches effective intra-lesional concentrations more rapidly than B, is expected to combine with nitroimidazoles and Q more effectively and shorten treatment in the clinic (65). Because of the limited head-to-head comparisons of D and P (10), it remains unclear which nitroimidazole is best for TB treatment. We did not find a significant difference between the two drugs when they were administered as single agents. However, less frequent dosing of P (once daily versus twice daily for D) and the higher manufacturing costs for D suggest practical and economic advantages for P administration (10). It is possible that D could be used in cases when *M. tuberculosis* isolates are resistant to P, as resistance patterns in deazaflavin-dependent nitroreductase do not fully overlap for these nitroimidazoles (66–68).

The DBQ combination regimen in the marmoset was not statistically different from the HRZE regimen in terms of pathology, inflammation, and sample culture positivity. A Phase 2 DBQ trial (39) in drug-susceptible pulmonary TB patients was recently published (69). High rates of sputum culture conversion were achieved at the end of the 4-month treatment with the three-drug combination. The regimen was generally well tolerated. The control arm that received 6 months of HRZE also achieved a high rate of sputum culture conversion by the end of treatment. However, at the exploratory endpoint of 12-month post-randomization relapse, the rate in the DBQ arm was numerically higher than in the HRZE arm (69). As with this marmoset study, the DBQ clinical trial was not powered to compare the DBQ and the HRZE regimen regarding efficacy, but the trial’s relapse results suggest that additional drugs need to be added to the three-drug DBQ core regimen to outperform HRZE and to shorten treatment duration.

Human-like exposures of D and B were achieved in this study, although in the marmoset, the abundance of B-M2 required it to be accounted for in the overall exposure to B (Table 1 and Fig. S1) (32, 35, 37). The appropriate human dose for Q is still being determined in clinical trials (39). In this study, after a single oral dose of Q, marmosets had a *C*_max_ of 0.22 ± 0.09 µg/mL (mean ± SD) and an AUC_(0–24 h)_ of 3.7 ± 0.9 µg · h/mL, approximating exposures observed in humans following a single dose of 60–90 mg of Q (38). After 14 days of once-daily dosing, the marmoset AUC_(0–24 h)_ of 3.7 ± 2.1 µg · h/mL which remained in the range of clinical Q exposures reported in patients receiving 30–90 mg daily in the 14-day early bactericidal activity (EBA) study. By 28 days, however, Q exposure had increased substantially. In animals receiving Q alone or Q in combination with D or B, Q exposure was approximately threefold higher than clinical exposure. In the DBQ combination, Q exposure was approximately 10- to 15-fold higher than clinical exposure. The increase in Q exposure after multiple days of Q dosing alone suggests time-dependent accumulation, potentially due to saturation of clearance mechanisms (70). The greater increase observed with the DBQ regimen may reflect a combination of CYP450 inhibition by ABT and additional drug–drug interactions among the coadministered agents (38, 70), possibly reflecting marmoset-specific drug disposition. Despite these higher exposures, we did not observe a Q exposure–response relationship at the dose levels used in this study. This finding is consistent with clinical data suggesting that Q efficacy may plateau at the 30 mg daily dose, as prior studies did not detect clear efficacy differences between 30 and 90 mg dose levels (35, 39). Comparable accumulation of Q was not observed in humans receiving these drug combinations in either the 14-day EBA trial (41) or in the 4-month DBQ trial (Y Liu, unpublished data), supporting the interpretation that the magnitude of Q overexposure observed in marmosets may be species- and regimen-context-specific.

This study has several limitations. First, the number of animals per treatment group was small, and a priori sample size calculation was not performed. Although cavities and other lesion types were analyzed as the biologically relevant unit, consistent with established nonhuman primate and rabbit tuberculosis models, multiple lesions from the same animal are not fully independent, especially under drug pressure. A sensitivity analysis using a linear mixed-effects model demonstrated that accounting for animal-level clustering produced more conservative statistical inference for some comparisons, yet the overall pattern of treatment effects was similar to that observed in the primary lesion-level analyses. An additional limitation of this study is that the treatment groups were not powered to determine the inferiority between the DBQ and HRZE regimens. In addition, the majority of the HRZE study animals were not conducted concurrently with the DBQ study animals. Because of the intensity with which each marmoset was examined and the resources required to handle these NHPs, relapse studies have not been conducted. It is possible that we overestimated the activity of Q because drug–drug interactions increased exposure to the agent in this study, but data from clinical trials and this study indicate a likely efficacy plateau at the human 30 mg daily dose level, as discussed in the previous paragraph (38, 69). The potency of D against nonreplicating persisters in caseum could not be evaluated using the standard protocol due to rapid albumin-mediated degradation (50). By modifying the caseum assay protocol and providing daily replenishment of D, which can be considered as mirroring daily dosing in patients, we confirmed that D displays bactericidal activity against caseum *M. tuberculosis*. Finally, this study employed ABT to inhibit CYP-mediated metabolism and achieve target bedaquiline exposures in marmosets. As ABT is a non-specific CYP inhibitor and is not co-administered in clinical practice, it may affect the metabolism of co-administered agents as demonstrated for Q and represents a limitation when extrapolating these findings to humans.

In summary, Q had similar pharmacokinetic profiles in humans and marmosets when administered as a single agent for 14 days. It showed significant bactericidal activity alone and in combination with B and D at human-like exposure levels of these agents. Moreover, Q contributes to lesion sterilization despite its inactivity against non-replicating persistent MTB in the caseum model, confirming the utility of the DprE1-DprE2 complex as an *M. tuberculosis* drug target (9). Finally, in our preliminary comparison, the DBQ combination was not different from HRZE in terms of the reduction of lung lesion volume, FDG uptake, or bacterial CFU in cavitary lesions. Our data suggest comparable activity between D and P and superior activity of B and support the further development of Q as a new TB drug.

## MATERIALS AND METHODS

### Antibiotic formulations and pharmacokinetic studies

Delamanid hot-melt granules, spray-dried formulations, and quabodepistat powder were provided under a research agreement with Otsuka Pharmaceutical Co., Japan. ABT was formulated in 10% water, 90% suspension syrup (1:1 OraPlus/OraSweet and 2% (vol/vol) flavor, Padagis Inc., Allegan, MI). Delamanid was formulated in 20% (wt/vol) Captisol (Ligand Pharmaceuticals, San Diego, CA), 50% (vol/vol) OraSweet, 49% (vol/vol) water-saccharin (20% wt/vol), 1% vol/vol flavor, and pH adjusted to 3 using 1M citric acid. Bedaquiline (Sirturo, 100 mg) was mortar/pestle ground to a fine powder and formulated in 50% (vol/vol) OraPlus, 48% (vol/vol) OraSweet, and 2% (vol/vol) flavor. Quabodepistat was formulated in 50% (vol/vol) OraPlus, 48% (vol/vol) OraSweet, and 2% (vol/vol) flavor. When combining agents, 7- or 14-day dosing studies were performed to confirm the combination tolerability in uninfected marmosets. Formulations of the agents at the doses described in Table 1 were presented to hand-caught, awake, and restrained marmosets at 1 mL/kg or 2 mL/kg for delamanid orally in 1 mL syringes (no needle), followed by a favored treat (e.g., cereal pieces or marshmallows). The modeling and dose selection of bedaquiline have been previously described in detail (28). ABT was administered daily 30 min prior to any suspension containing bedaquiline. Delamanid was administered at least 30 min after bedaquiline in the DB and DBQ combinations. ABT’s effect on Q AUC was estimated in naïve marmosets given ABT 1 day and 30 min before daily Q dosing (7.5 mg/kg) for 14 days (*n* = 4), compared with marmosets given Q alone daily (*n* = 4). Blood samples were collected from each animal after the first and 14th Q doses.

### Pharmacokinetic analysis and bioanalytical methods

Small blood samples were drawn after dosing at intervals (e.g., 30 min, 1, 2, 4, 8, 12, and 24 h) into lithium heparin-containing microtainers (BD, Lakes, NJ) and immediately centrifuged for 5 min at 8,000 × *g* to remove cellular constituents, and the resulting plasma was frozen for later drug analysis. Samples were collected from a minimum of three naïve marmosets after a single dose and 2 weeks (3 weeks for B) of daily dosing for single-dose and steady-state pharmacokinetic parameter estimation. Blood samples were collected from all *M. tuberculosis*-infected marmosets after 2, 5, and 7 weeks of treatment at the estimated peak and trough times for each agent. Plasma concentrations of the agents were determined using previously developed high-performance liquid chromatography-tandem mass spectrometry bioanalytical methods (17, 71, 72). The area under the curve from 0 to 24 h (AUC_0–24 h_) was determined using standard non-compartmental techniques.

### Monitoring of disease by PET/CT

For PET/CT, commercial FDG (2 mCi/kg; Cardinal Health, Beltsville, MD, USA) was administered intravenously, flushed with ~ 2 mL of saline, and the animals were maintained under sedation for 1 h before the PET scan was collected for a minimum of 15 min/bed position. PET/CT imaging was performed either with a Siemens microPET scanner (Siemens Preclinical Solutions, Knoxville, TN, USA) paired with an 8-slice CereTom helical CT scanner (CereTom, NeuroLogica, Danvers, MA) using a shared animal bed for image alignment (43) or with an LFER-150 PET/CT scanner acquired after support for the original systems ended. Before using the LFER scanner, CT acquisition settings were cross-calibrated to the CereTom using a custom Mediso soft-tissue phantom to identify the protocol that best reproduced expected HU values. The optimized protocol was further evaluated in *M. tuberculosis–*infected rhesus macaques and rabbits scanned on both systems on the same day or within the same week, respectively, by comparing identical lung lesions using matched regions of interest. In paired animal scans, scanner-related HU differences were approximately 50 HU, with the largest differences in the lower-attenuation range, approximately −500 to −101 HU. Although lesion thresholds captured the full −500 to 200 HU range, between-animal and between-group analyses focused primarily on the −100 to 200 HU (hard disease) range that was similar between scanners.

Images were collected beginning at the base of the animal’s head, through the lung, and into the abdominal cavity (250 mm scan) with respiratory gating on the LFER (73). Histograms of the emission list-mode data were processed and corrected, and CT and PET images were fused. PET/CT files were further co-registered, and serial scans were aligned using MIM Maestro (v. 7.0, MIM Software Inc., Cleveland, Ohio, USA). Each lung abnormality (not observed in the pre-infection scan) was assigned a number and tracked in a three-dimensional volume of interest (VOI) through each of the six serial scans, which sometimes expanded and merged during the infection progression before typically shrinking during drug treatment. Using these preliminary findings, three readers prepared the VOIs for each set of animal lesions, and the VOI numerical data extracted included features such as the CT Hounsfield Unit max, mean, minimum, HU standard deviation, the radiodense volumes (hard or −100 to 200 HU; soft or −500 to −101 HU), and measures of the FDG uptake/mL (referred to as TGA) and mean and max SUV. The SUV-based measurements were both corrected for partial volume effects and normalized for day-to-day variability in FDG uptake by dividing the SUV of the VOI by that of a muscle VOI as described previously (43, 74, 75) so that FDG uptake can be compared across time points and machines. The three readings for each feature were averaged and assessed for changes in the subsequent scans (30).

### Infection and evaluation of drug efficacy

Marmosets were infected with *M. tuberculosis* strain H37Rv using a nose-only aerosol generated in a BANG nebulizer through an aerosol exposure system (CH Technologies, Westwood, NJ). *M. tuberculosis* exposure in this experiment (composed of eight sets of six or eight animals) resulted in an average of 14 lesions/marmosets (SD ± 6), as assessed by PET/CT at 5 weeks post-infection in this study. Marmosets were stratified by pre-treatment lesion volume prior to drug group assignment to distribute disease burdens equally among the treatment groups (28). Drug treatment was started after a ~7 weeks PI FDG PET/CT and continued for 8 weeks with PET/CT imaging to monitor the response to therapy, as described previously (27, 30). The treatments were distributed among 2–3 infection sets until at least five animals had been treated with each agent or regimen. PET/CT readers and scientific staff performing the necropsy and microbiological enumeration were blind to treatment group assignment until all data were extracted or tabulated. At necropsy, all PET/CT identified lung lesions, pieces of apparently normal lung, the paratracheal, carinal, hilar, and right axillary lymph nodes, and samples from spleen, liver, and kidney were weighed, homogenized, and plated in triplicate onto M7H11/OADC agar supplemented with albumin, oleic acid, dextrose, and activated charcoal powder (0.4% wt/vol) to enumerate bacterial CFU per lesion or organ. In addition, portions of the lung lesions and tissues were processed for histology and stained with hematoxylin and eosin and Masson’s trichrome, as previously described (43). The CFU data and histological assessment for each tracked lesion or structure were merged with the PET/CT lesion data using the prepared necropsy maps to evaluate the response of the individual lesion types during treatment, as previously described (27, 30). For individual lesion CFU and histological analysis, if separate structures could be distinguished within a radiologic VOI at necropsy, the structures were evaluated separately, where possible, to distinguish the differences in lesion types more accurately since the features were evaluated separately.

### Statistical analysis

For the CFU data, a log10 (*x* + 1) transformation was implemented so that zeroes were not excluded from the graphs or analyses. The limit of detection for a sample was plotted when no CFUs were detected. Statistical analyses were performed using GraphPad Prism 11 (GraphPad Software, San Diego, CA) or R, and multiple groups were compared using one-way ANOVA with Holm-Sidak’s multiple comparisons test unless specifically listed otherwise in the corresponding figure legend. As a sensitivity analysis, lesion-level cavity CFU data were also analyzed using a linear mixed-effects model using R in RStudio (Version 2026.01.2+418) with treatment group as a fixed effect and animal as a random intercept to account for clustering of lesions within individual animals. *P* values for the planned comparisons were adjusted for multiple testing using the Holm procedure. The ICC was calculated to quantify the degree of within-animal clustering. When two groups were compared, an unpaired *t*-test was used. Group means for marmoset disease volume treated with or without quabodepistat were compared by Welch’s *t*-test. Fisher’s exact test was used to compare the categorical data. Statistical significance was set at *P* < 0.05. Error bars represent the standard deviation of the mean, and the following symbols were used for *P* < 0.033 (*), <0.002 (**), <0.001 (***), and <0.0001 (****) unless otherwise noted.

## Data Availability

Marmoset PET/CT scans are available at https://accessclinicaldata.niaid.nih.gov/study-viewer/clinical_trials/MARM-TB-DRUG under the title of Tuberculosis Infected Marmosets. Assess requires registration on the site and submission of a request form found there. Other data have been included in the manuscript and in the supplemental material.

## SUPPLEMENTAL MATERIAL

Supplemental material aac.00280-26-s0001.pdf Supplemental methods; Fig. S1 to S9; Tables S1 to S4.

ASM does not own the copyrights to Supplemental Material that may be linked to, or accessed through, an article. The authors have granted ASM a non-exclusive, world-wide license to publish the Supplemental Material files. Please contact the corresponding author directly for reuse.
