# Supplementary material for: Efficacy of quabodepistat alone and in combination with delamanid and bedaquiline against Mycobacterium tuberculosis in an NHP model evaluated by [18F] FDG PET/CT and bacterial burden
Source: Antimicrob Agents Chemother. 2026 Sep 16;70(10):e00280-26. doi: 10.1128/aac.00280-26 (PMC13688883; doi:10.1128/aac.00280-26)
Supplement: Supplementary file 1 [file aac.00280-26-s0001.pdf]

Efficacy of quabodepistat alone and in combination with delamanid and bedaquiline against *Mycobacterium tuberculosis* in an NHP model evaluated by [^{18}F] FDG PET/CT and bacterial burden.

Via L.E.[1,2#], J Sarathy[3,4], D.M. Weiner[1,2], T. Greenstein[5], M. Zimmerman[3], A. Abdi[2], J. Fleegle[2], F. Gomez[2], F. Kaya[3], M.K. Piazza[2], A. Vatthauer[2], B.Y. Sloan[2], K.M. Repoli[2], A.M. Walker[2], M.J. Woodcock[2], O. Egbelowo[6], K. Gausi[6], H.I.M. Boshoff[1], Y. Liu[7], D. Chadalavada[8], B.B. Aldridge[5], V. Dartois[3,4], C.E. Barry 3[rd,1,9]

Supplementary Methods and Results

MIC and resistance determination of M. tuberculosis from tissue samples.

The minimum inhibitory concentrations (MICs) presented in Table 1 are cited from literature mentioned. To confirm, we determined the MICs of the M. tuberculosis H37Rv clone used in this study using liquid M7H9/ADC broth with 0.05% Tyloxapol in 96 well plates as described in Wang et al., 2021. The MICs were within the published ranges for each agent. For pretomanid, the H37Rv clone use here had an MIC between 0.09 and 0.16 ug/mL.

Development of resistance in vivo was assessed by either picking 5-10 of the colonies growing out from lesion samples on M7H11 agar plates with charcoal and determining the MIC or by plating diluted lesion homogenates onto M7H11 agar containing 5x and 20X MIC concentration of the agents dissolved in DMSO and comparing the number of colonies resulting to those on DMSO containing agar. The agar plates used contained either 0.75 mg/L or 3 mg/L bedaquiline; 0.005 mg/L or 0.02 mg/L quabodepistat; 0.1 mg/L or 0.4 mg/L delamanid or 1 mg/L or 4 mg/L pretomanid.

No resistance to the agents other than pretomanid was detected. One marmoset had at least 4 cavitary lesions with resistant *M. tuberculosis* colonies with MICs of 3.5 to 10 ug/mL pretomanid and its tissue samples grew bacterial colonies on M7H11 agar plates containing 1 mg/L and 4 mg/L pretomanid. An additional animal had 1 cavitary lesion that also grew colonies on the pretomanid containing agar. These lesions are represented as open symbols in Fig. S3G.

Dosing and MIC of ABT

To inhibit metabolism of bedaquiline (B) in the marmoset so we could more closely mimic *M. tuberculosis* exposure in humans, 1-aminobenzotriazole (ABT) was administered at 20 mg/kg daily.

ABT had a peak plasma concentration of 12.9 µg/mL (± 1.7 µg/mL SD, n=4) at 1 h in marmosets after 4 days of dosing. The MIC of ABT for M. tuberculosis was > 100 µg/mL after 1 week in microtiter plates with M7H9/glycerol/OADC/Tween 80 at 37 C .

Pharmacokinetics parameters of pretomanid. P as a single drug has been previously described in marmosets in Budek et al, 2024 and in Greenstein et al, 2026.

Supplemental statistical methods.

Exploratory exposure–response analysis using Spearman rank correlation, ordinary least squares regression, and regimen-adjusted multivariable linear regression, and multifactor linear models

Exposure–response relationships between drug exposure and microbiologic outcomes were evaluated using animal-level data. Lesion-level CFU measures were summarized for each animal as mean and median lesion CFU; individual lesions were not treated as independent experimental units. Exposure metrics included drug Q AUC_{0-24}, D AUC_{0-24}, B+M2 AUC_{0-24}, and B+M2 C_{avg}. Spearman rank correlation was used to assess monotonic associations between exposure and outcome. Ordinary least squares regression was used to evaluate unadjusted linear relationships, and multivariable linear regression was used to adjust for treatment regimen, with regimen included as a categorical covariate. Exposure metrics were $\log_{10}$ transformed for regression analyses. All tests were two-sided, with $p<0.05$ considered statistically significant. The results can be found in Fig. S7 and Table S4.

Exploratory component-level analyses: Each animal was coded according to the presence or absence of B, D, and Q, and animal-level outcomes were analyzed using three-way factorial linear regression models with B, D, and Q as categorical fixed effects on change in disease volume (Fig S5) or culture positivity (Fig S6) as dependent variables. P values for the component-level comparisons were adjusted using the Holm method. Analyses were performed in R, and adjusted p values were calculated using the p.adjust (method = “holm”) function. The results of the analyses are presented in Fig. S5 and S6.

FIG S1. Performance of the human-like dose of bedaquiline compared with an initial higher dose. (A) Table showing the initial high dose and a subsequent lower dose of bedaquiline administered to marmosets 30 min after administration of 20 mg/kg 1-aminobenzotriazole daily to reduce metabolism and the resulting concentration average (Cavg) of parent and desmethyl-metabolite observed +/- standard deviation (n= 6 or more measurements; 2 per marmoset). Modeling of the lower 8 mg/kg (mpk) x 14 days; 6 mg/kg 3 x wk. dose has been described (Greenstein, Sci Tans Med, 2026). The lower dose of bedaquiline achieved a steady state Cavg like the human dose (Svensson et al., 2014) and was used for all combinations, but it was not maximally effective as shown by the higher remaining total lung bacteria in the estimation plot (B, p=0.03 unpaired T test), and the higher culture positivity rates of lung lesions (C), yet the residual pathology measure by CT (D) was not significantly different between the doses although the sample size was small. P values in C ** 0.002, *** 0.0002, **** <0.0001 in by ANOVA HS-MCT.

A

BDQ administration (mg/kg or mpk) Daily ABT 20 mg/kg	BDQ $C_{(avg)}$ ng/mL	Des-BDQ $C_{(avg)}$ ng/mL
20 mpk x 15 Days/20 mpk 3 x wk for 6 wks	4668 +/- 1493	3814 +/- 796
8 mpk x 15 Days/6 mpk 3 x wk for 6 wks	1365 +/- 373	2608 +/- 576

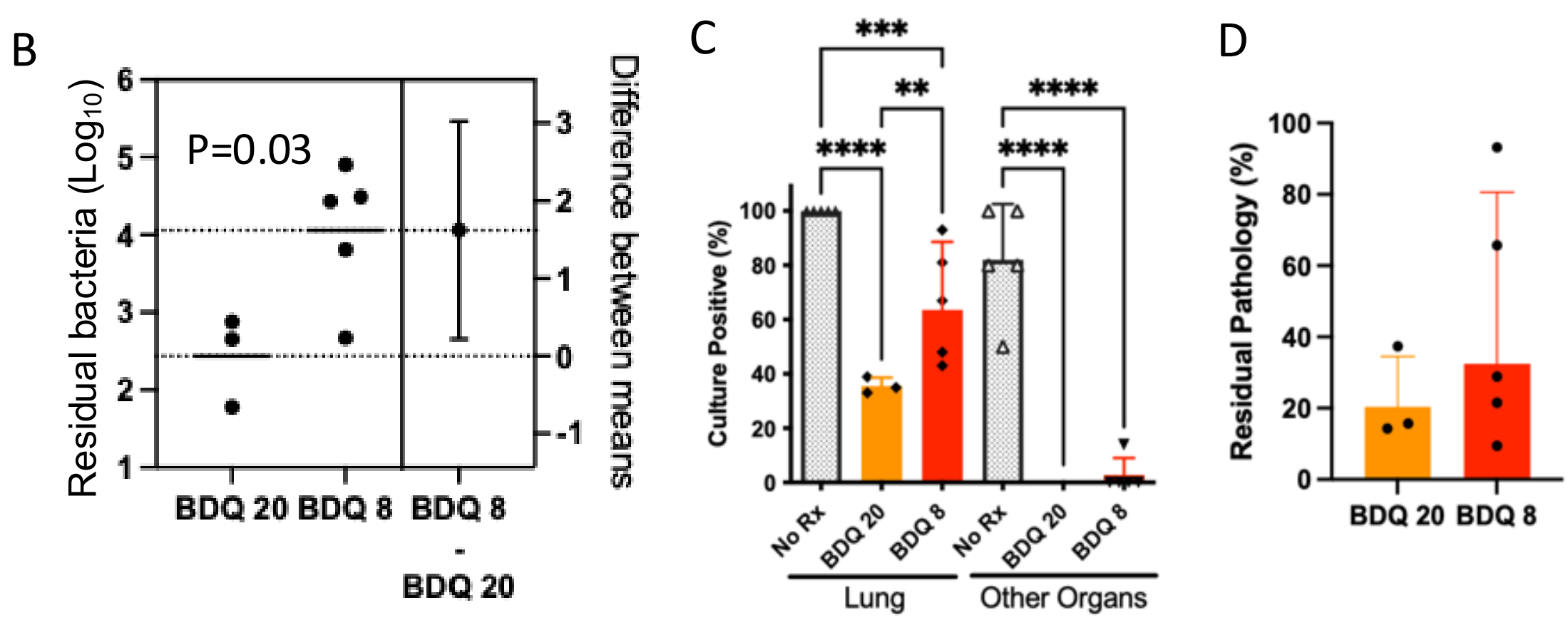

FIG S2. Quabodepistat plasma concentration – time profile with or without ABT (A) Curve after 7.5 mg/kg Q dose +/- ABT with AUCs below. (B) Curve resulting from IV dose of 0.5 mg/kg Q with parameters shown below. Lines are means, error bars St error of the mean.

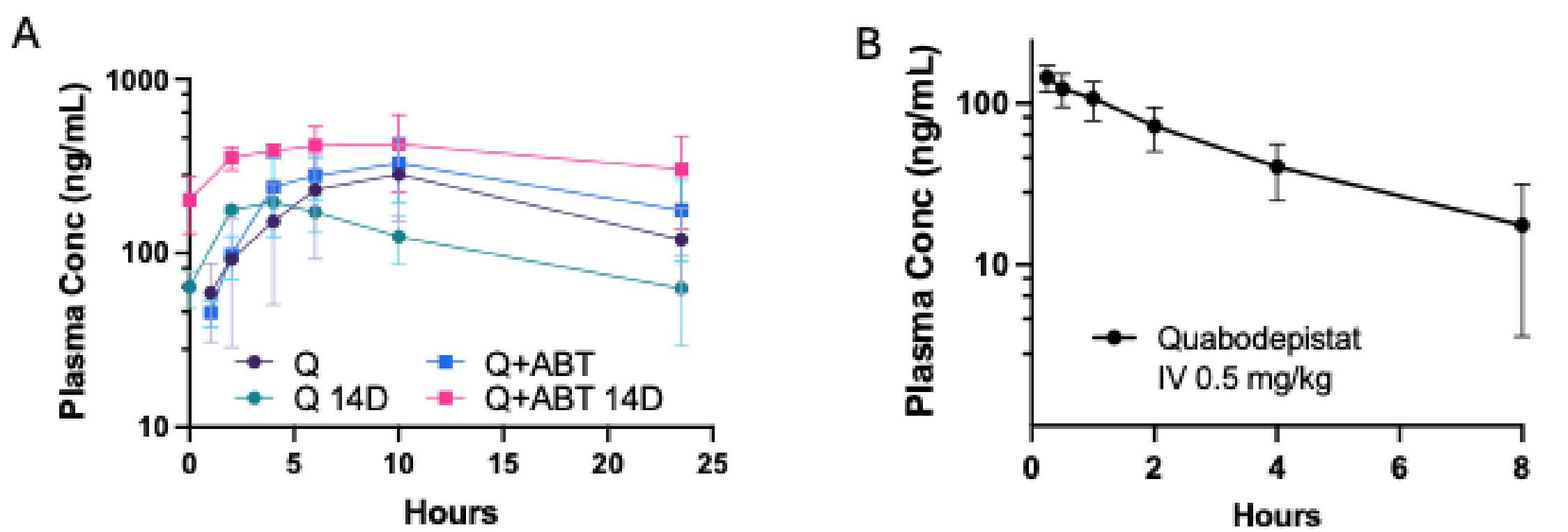

Oral 7.5 mg/kg	AUC Single Dose (ng*h/mL)+/- SE	AUC 14 doses (ng*h/mL)+/- SE
Q	4487 +/- 1070	2839 +/- 369
Q+ABT	5565 +/- 1120	8681 +/- 1828

Single IV dose	
AUC[0-∞] (ng*h/mL)	393 +/- 412 SD
Kel (h^1)	0.304
IV Half Life (h)	2.28 +/- 2 SD
Vd (L/Kg)	4.18 +/- 0.5 SD
Clearance (mL/kg*min)	21.2 +/- 8 SD

.

Table S1. Change in delamanid exposure [AUC 24h] with dosing of other agents

Doses	Alone (ng/mL)	with Q (ng/mL)	with B + ABT	with Q, B+ ABT,
1 dose	3,180			
7 to 14 doses	5,735		3,460	2,356
14 to 28 doses	6,053	7,236		
> 28 doses	4,916	1,781	3,782	2,634

Results are median concentrations (ng/mL) found in 3-5 infected marmosets after the indicated number of doses.

FIG S3. Delamanid and Pretomanid were not significantly different in activity as single drugs in the marmoset. Graphs depict the (A) Change in CT-based disease volume after 2 weeks treatment of with Delamanid (D) or pretomanid (P) where disease volume pretreatment is represented by 100%. Open symbols in A-G indicate the marmoset or lesions where resistance to P was detected by a > 4-fold increase in MIC or by colonies forming on agar plates containing 5X or 20X the P MIC. (B) Mean residual pathology percentage and (C) mean residual inflammation percentage after 2 months of drug administration measured from the final PET/CT scans as percentage of pathology or FDG uptake (SUVbw/mL/day), respectively. (D-E) Although both drugs reduced sample culture positivity (D, **, p<0.007 ANOVA HS-MCT), there was no difference in culture positivity rates of lung samples (D, p>0.905 ANOVA HS-MCT) or (E) extra- pulmonary samples (p>0.794 ANOVA HS-MCT) between the two nitroimidazoles. (F) Administration of both drugs reduced total lung CFU (***, p< 0.002 ANOVA HS-MCT), and (G) individual cavity CFU, (****, p< 0.0001 ANOVA HS-MCT), but there was no significant difference in the remaining bacterial burden between the two nitroimidazoles (p= 0.674 and p= 0.754, respectively). When the results from the animal with multiple resistant lesions (A-F) or the 5 cavities that were phenotypically P-resistant (G) were excluded from the statistical calculations, the two drugs remained insignificantly different in each comparison.

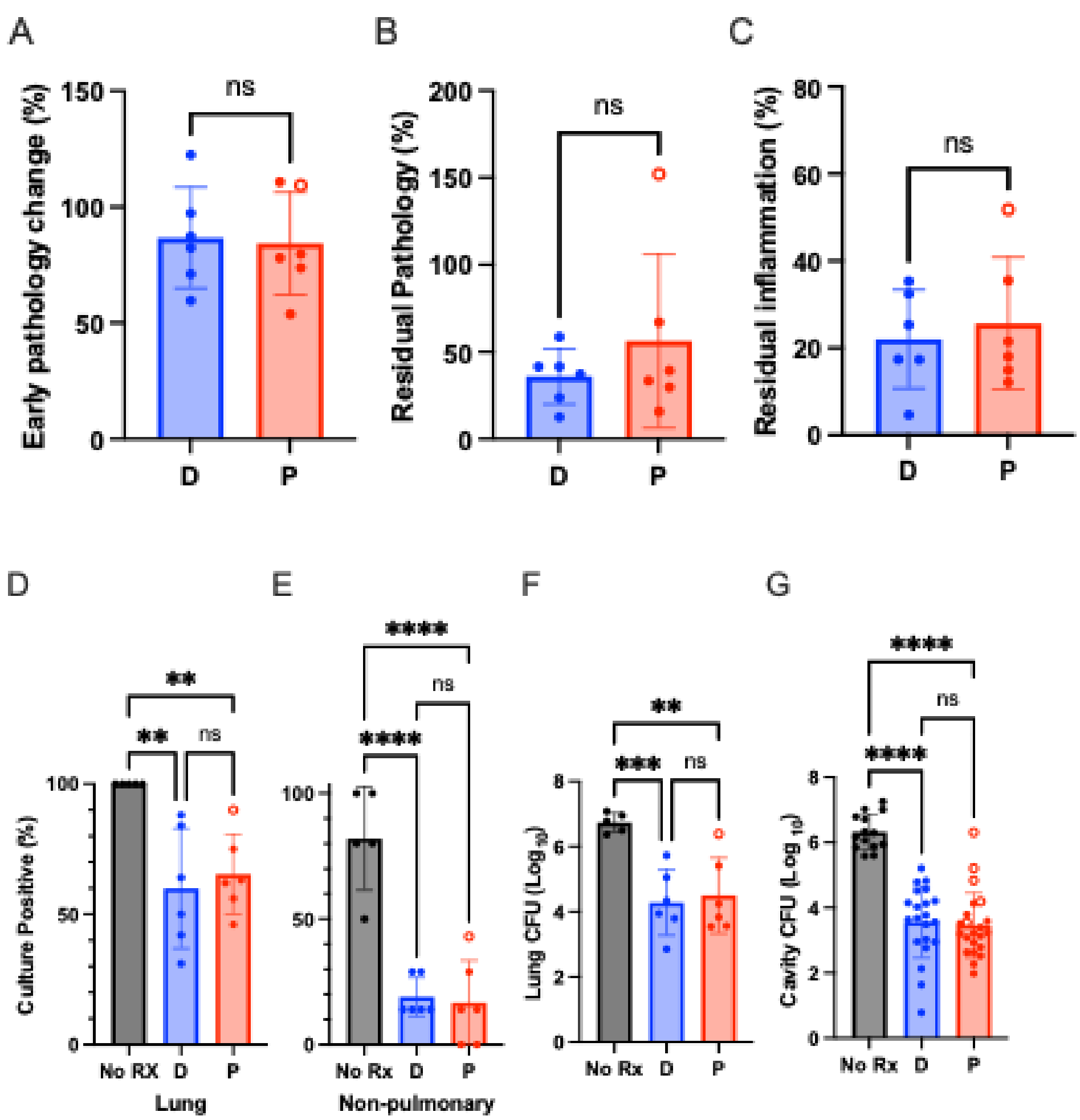

FIG S4. Illustration of the change in disease volume (CT) and FDG uptake (PET) in individual lesions by animal and by treatment group over time. Scans were collected beginning 30-34 days post infection (time point [TP] 1) and every two weeks there after (TP 2 - treatment initiation, TP 3 - 2 weeks of treatment; TP 4 - 1 month , TP 5 – 6 weeks; and TP 6 – 8 weeks). Treatment began after the second scan at TP2 and continued for 2 months (TPs 3-6). Pulmonary lesions were identified by comparison with each animal's pre-infection scan and tracked individually through the disease course. A) Change in hard (HU > -100) volume of pulmonary lesions in marmoset BQ05 treated with DBQ. Lesions > 0.05 mL at TP2 are shown. B) Total of the lesion's volume in each marmoset treated with DB over time. C) The mean and standard deviation (error bars) of each treatment group's marmoset total lesion volume over time. The same series of comparisons were constructed for FDG uptake measured from the PET scans. D) FDG total glycolytic activity uptake in ratio to the muscle uptake (TGA ratio; White et al., J Vis Exp, 2017) in marmoset BQ05's lesions shown in A . E) Sum of the lesion's FDG TGA ratio in each marmoset treated with DBQ. F) Mean and standard deviation (error bars) FDG uptake ratio in each treatment group of marmosets during observations. C and F share a legend shown on F. G) Distribution of total volume of lung lesions in marmosets assigned to the treatment arms measured at time point 2 just prior to treatment initiation. Each dot represents a marmoset randomized to the treatment group. The distribution of disease volume was not different among groups (p >0.95 ANOVA with HS-MCT post test). Error bars: A, B, D, E standard deviation of 3 readers measurements of the CT/PET scans. C, F, and G, standard deviation of the observations among the marmosets in each group.

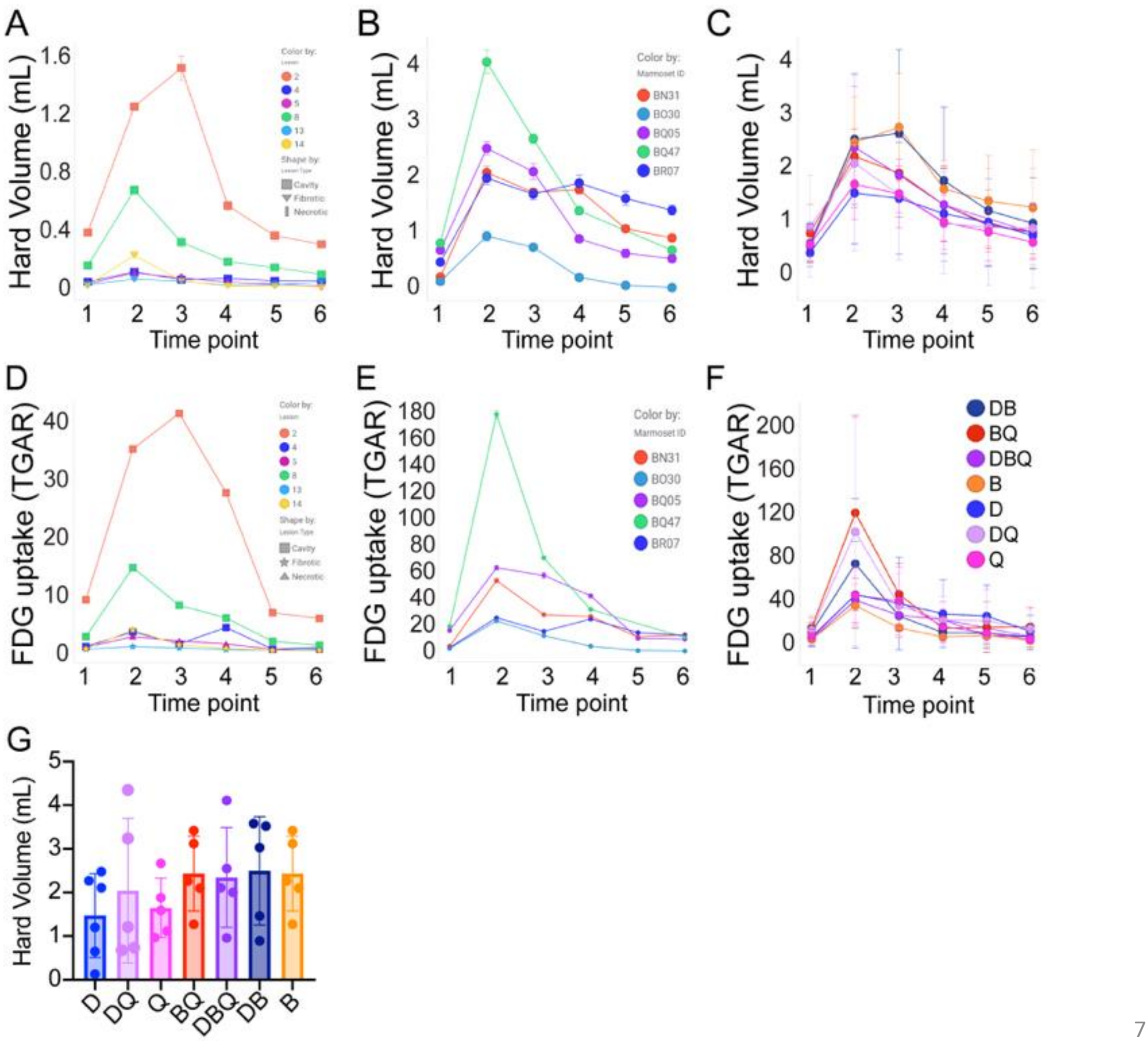

FIG S5. Treatment with B- or D-containing regimens was not associated with early change in marmoset lung disease volume compared with Q-containing regimens. In Fig. 1E, Q-containing regimens were associated with early change (reduction) in lung disease measured from serial CT scans collected pretreatment and after 2 weeks of treatment. By the same post hoc analysis method, bedaquiline- (A) or delamanid- (B) containing regimens were not significantly associated with change in disease volume (Welch's *t* test p=0.075 and p=0.142, respectively) with no B showing more reduction that B. To adjust for the fact that some animals were given 2 or 3 agents, a factorial linear model was used to evaluate if each component agent was associated with the change in disease volume after accounting for the whether the other agents were present. (C) Treated animals were coded according to regimen content and change in hard disease volume after two weeks was analyzed with B, D, and Q as fixed effects. After accounting for whether the regimen also contained B or D, the presence of Q was associated with greater early change toward reduced disease, confirming the observation in Fig 1E and Fig S5 A and B. Symbols show estimated associations for each drug component, with 95% confidence intervals as whiskers. Positive values indicate greater change toward resolving disease. P values shown were adjusted using the Holm method.

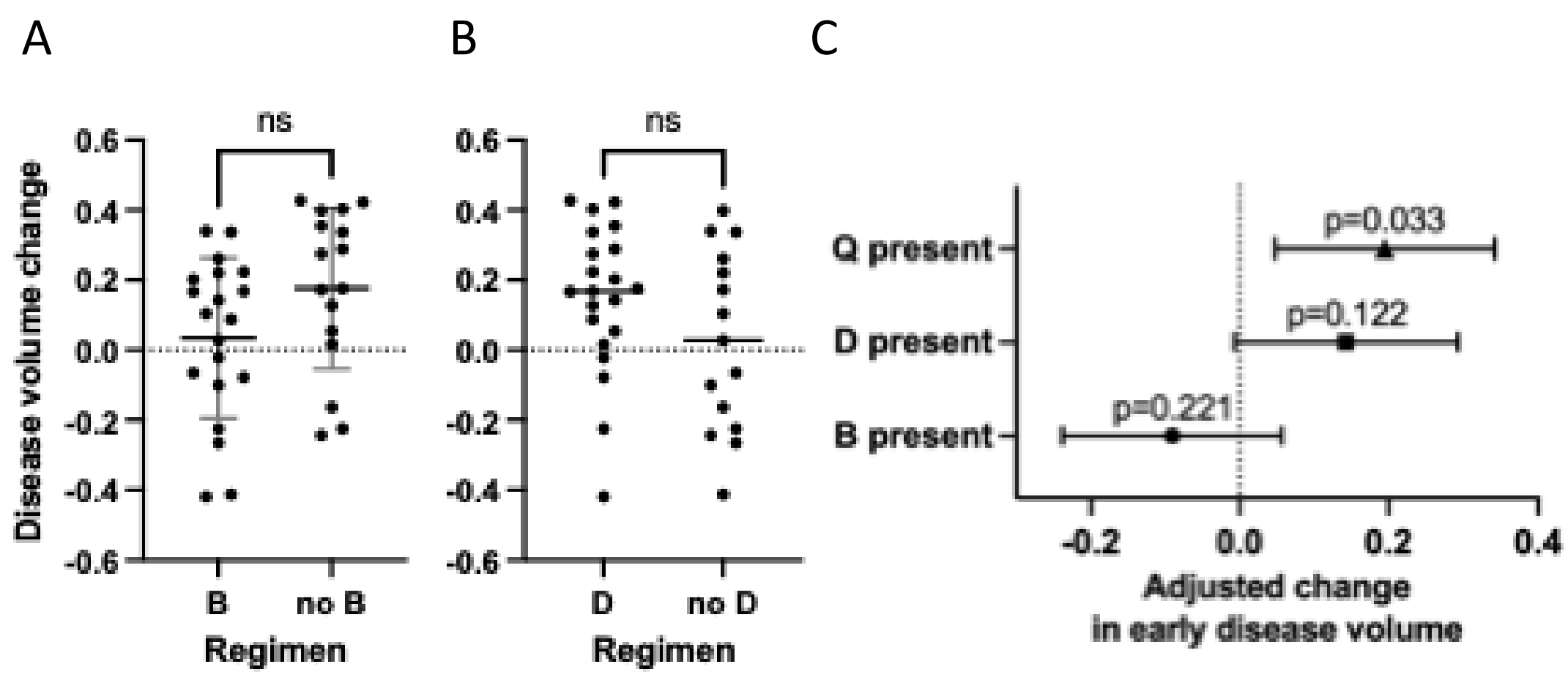

FIG S6. The presence of B, D, and Q were each associated with lower culture positivity of the marmoset lung and extrapulmonary samples. In Fig 2, the association of the individual regimens with culture positive tissue samples was displayed. Here in an exploratory post hoc analysis, the agent-associated lowering of lung sample culture positivity was explored. (A) Bedaquiline-containing regimens had lower rates of positive cultures (p=0.001, Welch's T test), but D- (B) and Q-(C) containing regimens were not significantly associated with the lower culture positivity in lung tissues using Welch's T test. (D) In a second analysis, a factorial linear model was used to evaluate if each agent was associated with reduced lung sample culture positivity after accounting for the whether the other agents were present. Each of the three agents was significantly associated with reduced culture positivity after adjusting p values by the Holm method. (E) The same methodology was applied to the animal's extrapulmonary tissue samples culture positivity and each of the three agents was significantly associated with reduced culture positivity. (D-E) Symbols show estimated associations for each drug component, with 95% confidence intervals represented as whiskers. Negative values indicate lower culture positivity of the samples. P values shown were adjusted using the Holm method.

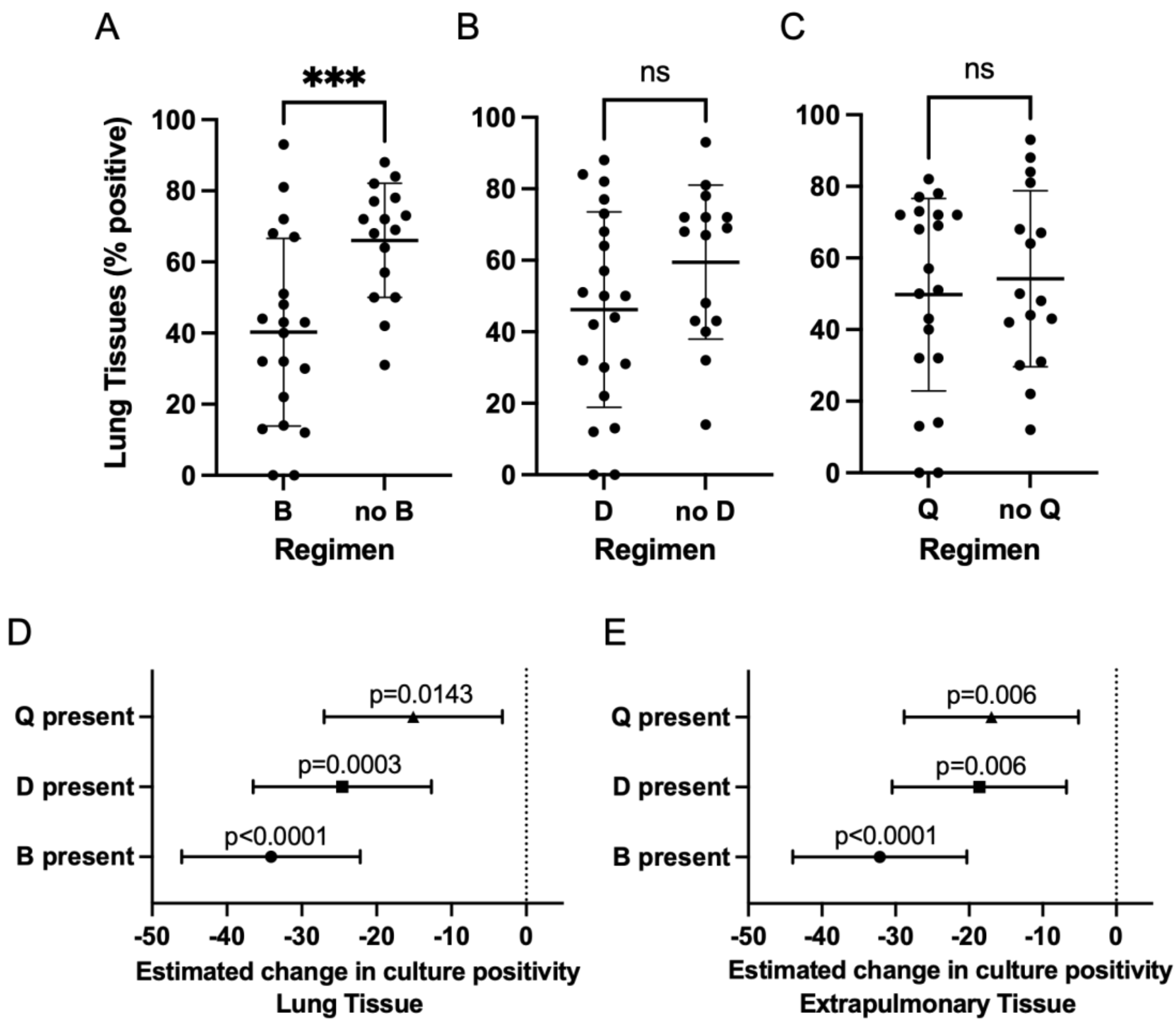

FIG S7. Dose-response curves for B, D and Q against nonreplicating, persistent *M. tuberculosis* bacilli resident in *ex vivo* rabbit caseum. (A-C) Bedaquiline (B) and quabodepistat (Q) were assessed as described previously (Sarathy et al., AAC, 2018); caseum specimens were exposed to single doses at desired concentrations for 7 or 14 days. Delamanid (D) dose-response was determined by replenishing the drug daily for 7 days to correct for rapid albumin-mediated metabolism (Sasahara et al., 2015). To correct for the different starting burdens of each experiment, bacterial killing is expressed as the change in the logarithmic functions of colony forming units (CFU) per ml of caseum homogenate relative to vehicle-treated controls (Δlog CFU/ml). Dots and error bars represent means and standard deviations of three technical replicates. (D-F) In comparison of final bacterial loads divided by lesion histological type, all three agents show evidence of reducing bacteria in all lesion types ($p < 0.001$ ANOVA with HS-MCT). In addition, in cavitary (D) and necrotic (E) lesions both Q and B were significantly more active than D in reducing lesion CFU ($p < 0.01$ ANOVA with HS-MCT) and in cellular/fibrotic lesions (F), B reduced bacteria significantly more than D ($p=0.006$ ANOVA with HS-MCT).

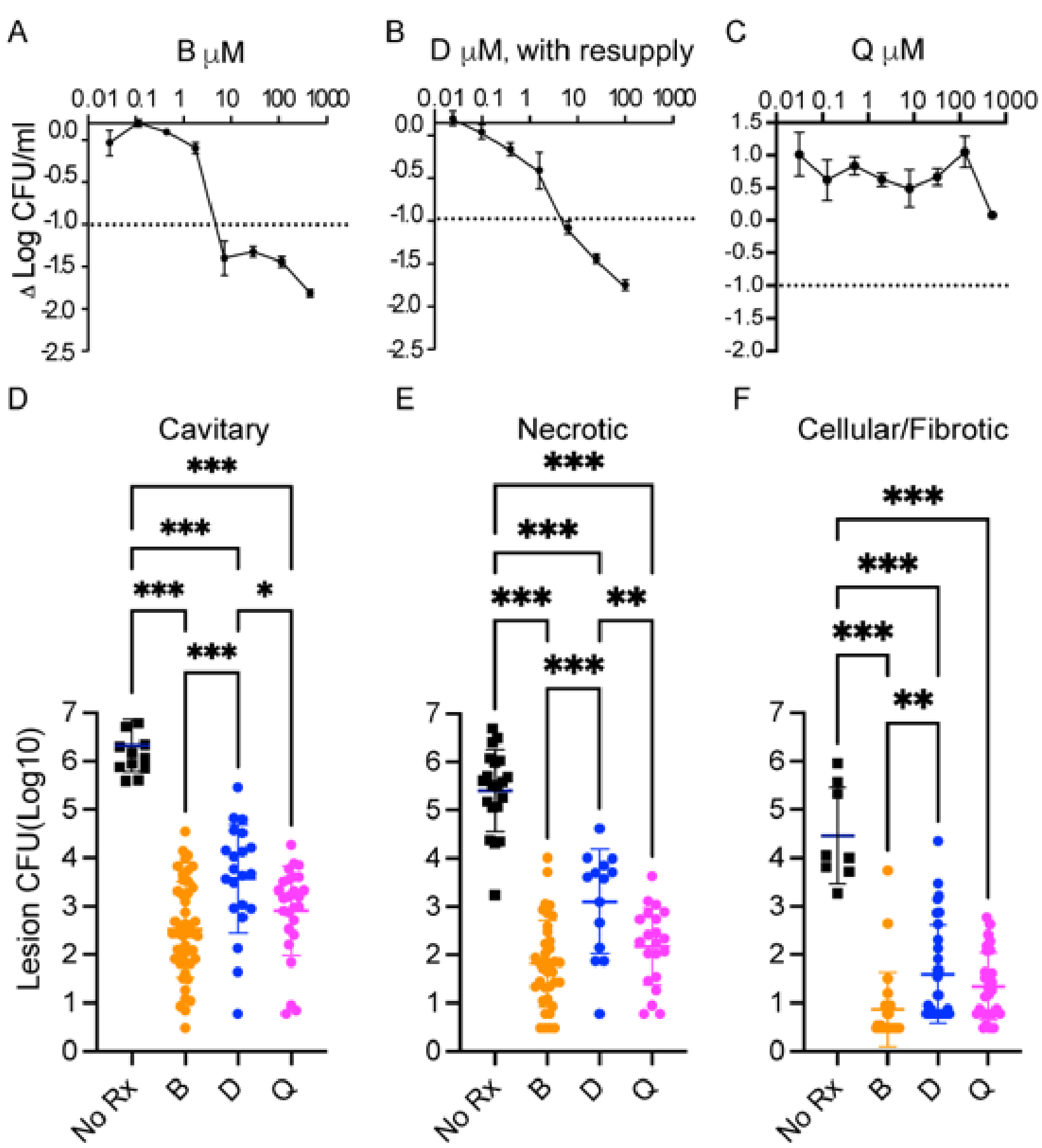

Table S2. Lesion distribution in the lesion-level analysis of Fig 3.

	Cavities				Necrotic				Fibrotic			
Group	Animals	Lesions	Mean	SD	Animals	Lesions	Mean	SD	Animals	Lesions	Mean	SD
B	5	46	10	10	5	37	7.4	1.7	5	25	5	2
D	4	22	5.5	3.7	6	14	2.3	2.1	6	34	6.2	2.6
Q	5	27	5.4	3	5	19	3.8	2.6	5	28	5.6	1.3
BD	3	11	3.7	2.9	5	24	4.8	4.4	5	46	9.2	3.4
BQ	4	22	5.5	2.4	4	18	4.5	2.7	5	26	5.2	3
DQ	5	10	2	1.2	4	14	3.3	1.5	5	21	4.2	2.3
DBQ	5	28	7	2.5	5	19	3.8	1.6	5	38	7.6	4.5

Table S3. Results of sensitivity analysis linear mixed-effects model

Lesion type	comparison	Esti-mate	SE	dF	T ratio	P value	Lower CL	Upper CL
cavities	DBQ vs B	1.0498	0.3889	14.6587	2.6995	0.0502	-0.1350	2.2346
cavities	DBQ vs BD	1.0731	0.4979	22.1585	2.1553	0.0703	-0.3691	2.5153
cavities	DBQ vs BQ	0.9596	0.4201	17.4236	2.2841	0.0703	-0.2901	2.2093
cavities	DBQ vs D	1.8774	0.4273	17.2956	4.3938	0.0023	0.6052	3.1497
cavities	DBQ vs DQ	1.6163	0.4718	31.2147	3.4259	0.0085	0.2873	2.9453
cavities	DBQ vs Q	1.4879	0.4015	17.3527	3.7054	0.0085	0.2928	2.6830
necrotic	DBQ vs B	0.7767	0.4180	21.3865	1.8583	0.2308	-0.4382	1.9917
necrotic	DBQ vs BD	0.4852	0.4516	24.6903	1.0743	0.2931	-0.8101	1.7804
necrotic	DBQ vs BQ	0.7969	0.4695	24.3218	1.6973	0.2308	-0.5514	2.1451
necrotic	DBQ vs D	1.8403	0.4696	32.6763	3.9190	0.0026	0.5215	3.1592
necrotic	DBQ vs DQ	1.4751	0.4775	28.1777	3.0891	0.0224	0.1201	2.8301
necrotic	DBQ vs Q	1.2276	0.4543	26.6934	2.7020	0.0473	-0.0670	2.5222
cell_fibrotic	DBQ vs B	0.3359	0.2762	19.1013	1.2159	0.7165	-0.4768	1.1485
cell_fibrotic	DBQ vs BD	0.1377	0.2616	15.5476	0.5265	1.0000	-0.6525	0.9279
cell_fibrotic	DBQ vs BQ	0.1613	0.2755	19.0860	0.5856	1.0000	-0.6492	0.9718
cell_fibrotic	DBQ vs D	1.1334	0.2612	18.0073	4.3387	0.0024	0.3595	1.9073
cell_fibrotic	DBQ vs DQ	0.9811	0.2847	20.4934	3.4462	0.0124	0.1499	1.8122
cell_fibrotic	DBQ vs Q	0.7097	0.2710	18.1937	2.6185	0.0692	-0.0923	1.5116

Lesion type	# lesions	# animals	Animal variance	Residual variance	Interclass Correlation Coefficient
cavities	166	31	0.2005	0.9330	0.1769
necrotic	145	34	0.2508	0.8580	0.2262
cell_fibrotic	218	36	0.1119	0.3968	0.2199

Figure S8. There was no clear relationship between Q or B exposure at steady state and remaining MTB bacteria in the lung. Points represent the sum of bacteria detected in lung lesions and sampled lung pieces of individual animals grouped by treatment regimen (color). (A) Although a significant inverse association is observed in pooled analyses of Q $AUC_{0\text{-}24}$ (ng*h/mL), separation by regimen demonstrates that this relationship is not consistent within treatment groups and is driven by differences between regimens rather than effect of Q exposure. (B) D AUC, (C) B+M2 AUC and (D) B+M2 average plasma concentration at steady state also were not associated with $\log_{10}$ CFU after adjustment for regimen. Similar results were seen for B alone.

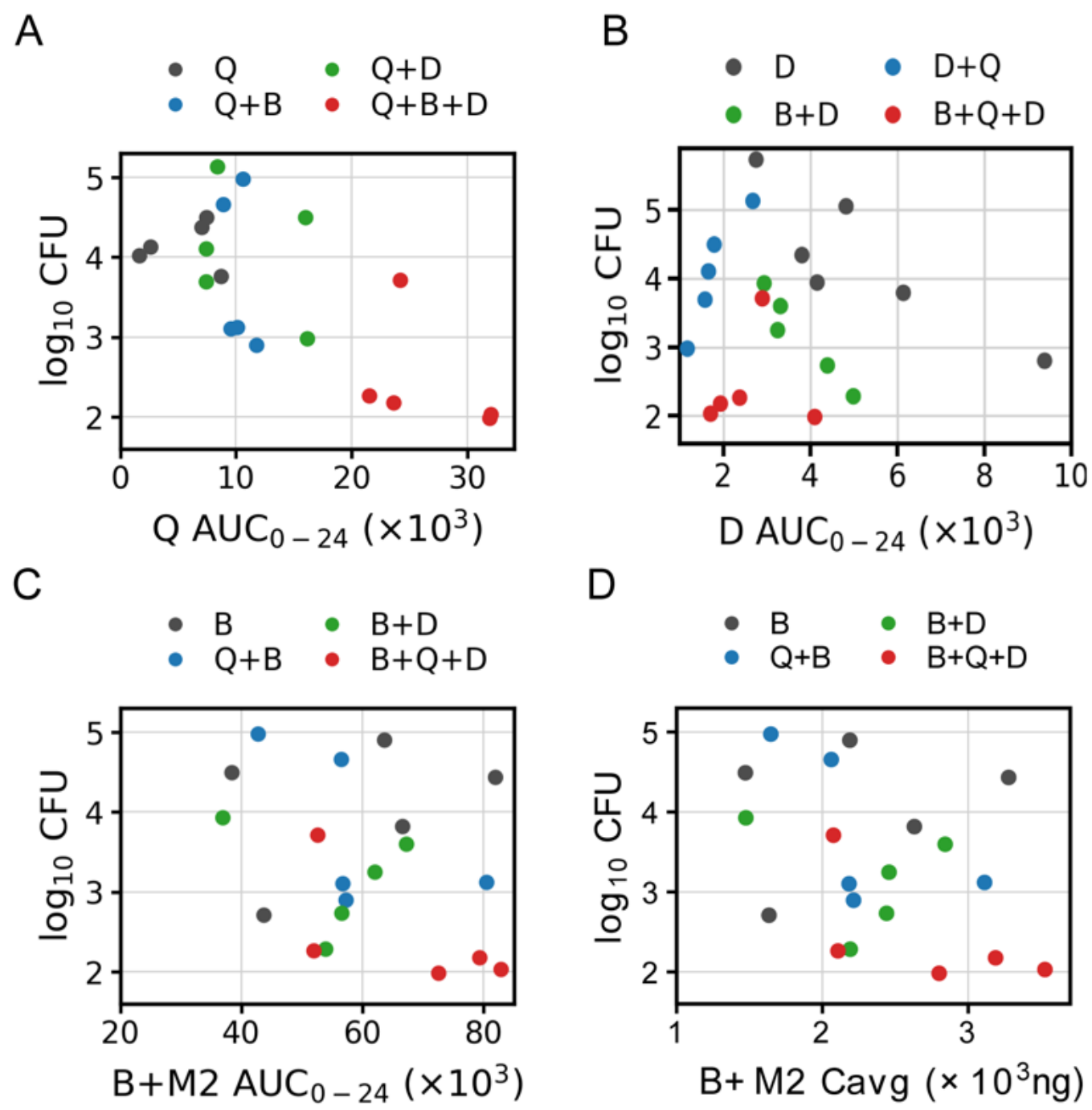

Table S4. Association of Q, D or B exposure and bacterial burden by regimen-adjusted multivariable linear regression.

Exposure metric	Outcome	Spearman rho (p)	OLS p-value	Adjusted p-value (regimen)	n
Q AUC0-24	Total CFU (log10 CFU)	-0.61 (0.004)	0.006	0.65	20
Q AUC0-24	Total CFU (raw)	-0.61 (0.004)	0.46	0.86	20
Q AUC0-24	Mean lesion CFU	-0.66 (0.001)	0.26	0.45	20
Q AUC0-24	Median lesion CFU	-0.55 (0.01)	0.54	0.46	20
D AUC0-24	Total CFU (log10 CFU)	-0.02 (0.915)	0.967	0.262	21
D AUC0-24	Total CFU (raw)	-0.02 (0.915)	0.925	0.142	21
D AUC0-24	Mean lesion CFU	-0.04 (0.871)	0.92	0.104	21
D AUC0-24	Median lesion CFU	-0.14 (0.549)	0.903	0.162	21
B+M2 AUC0-24	Total CFU (log10 CFU)	-0.31 (0.18)	0.16	0.32	20
B+M2 AUC0-24	Total CFU (raw)	-0.31 (0.18)	0.29	0.38	20
B+M2 AUC0-24	Mean lesion CFU	-0.38 (0.09)	0.34	0.46	20
B+M2 AUC0-24	Median lesion CFU	-0.26 (0.26)	0.05	0.06	20
B+M2 Cavg	Total CFU (log10 CFU)	-0.43 (0.06)	0.07	0.27	20
B+M2 Cavg	Total CFU (raw)	-0.43 (0.06)	0.13	0.28	20
B+M2 Cavg	Mean lesion CFU	-0.49 (0.03)	0.14	0.3	20
B+M2 Cavg	Median lesion CFU	-0.36 (0.12)	0.02	0.04	20

FIG S9. The DBQ combination was not significantly different in activity than the HRZE regimen. Graphs depict the (A) mean residual pathology and (B) mean residual inflammation measured as from the final PET/CT scan as volume of pathology or FDG uptake (TA in SUVbw/mL/day) respectively. (C) There was no significant difference in the rate of pathology change between the two regimens. (D) Administration of both regimens reduced total lung CFU (****, $p< 0.0001$ ANOVA HS-MCT), and (E) individual animal cavity mean CFU, (****, $p< 0.0001$ ANOVA HS-MCT), but there was no significant difference in remaining bacterial burden between the regimens ($p= 0.129$ and $p= 0.413$, respectively). Open symbols in A-E indicate marmosets where the bacterial load was below the limit of detection.

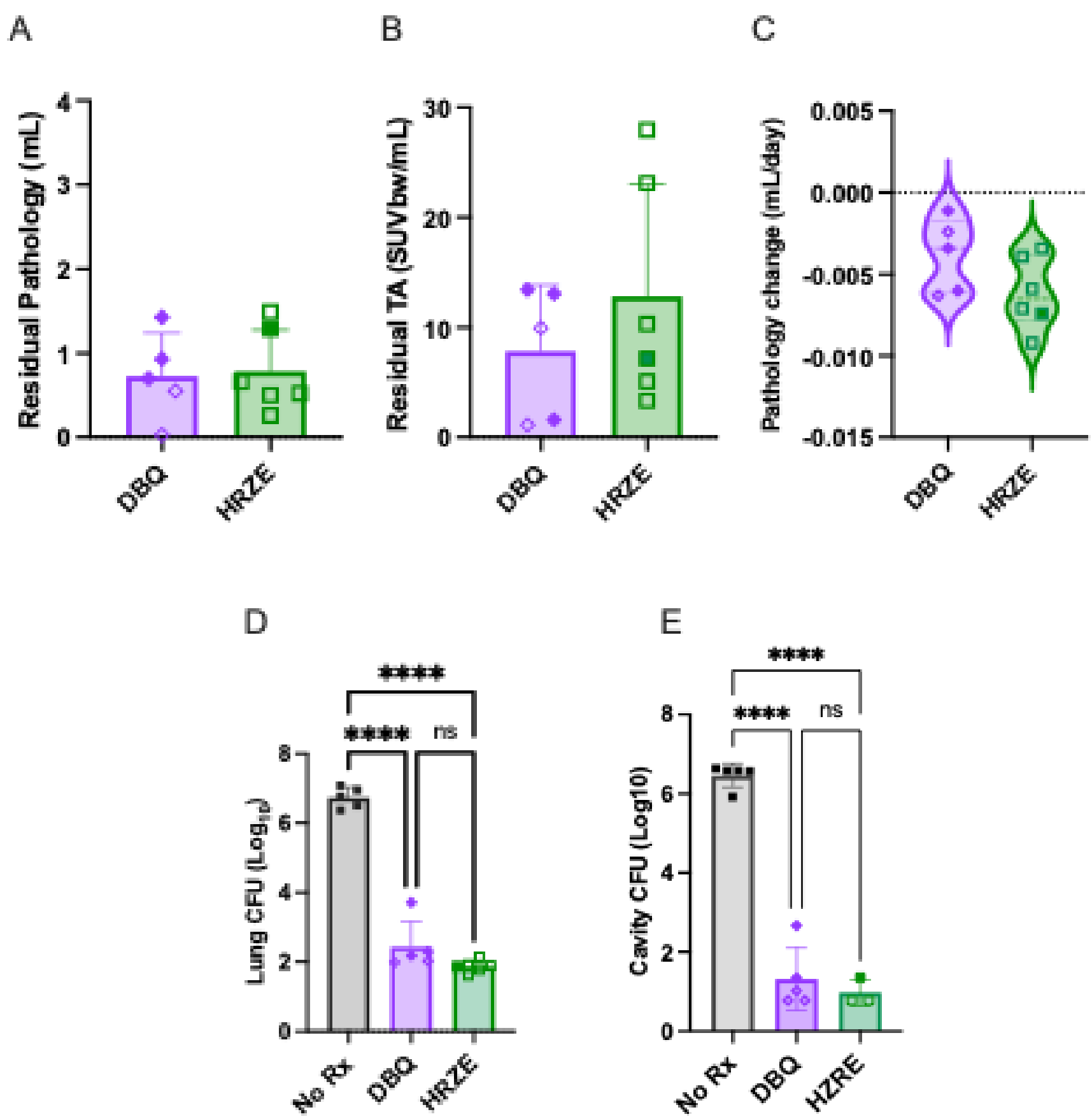

References in supplement:

Budak M, Via LE, Weiner DM, Barry CE 3rd, Nanda P, Michael G, Mdluli K, Kirschner D. A systematic efficacy analysis of tuberculosis treatment with BPaL-containing regimens using a multiscale modeling approach. CPT Pharmacometrics Syst Pharmacol. 2024 Apr;13(4):673-685. doi: 10.1002/psp4.13117.

Greenstein, T., L. E. Via, M. P. Moraes, D. M. Weiner, E. K. Dayao, A. M. Walker, A. Abdi, J. D. Fleegle, F. Gomez, K. M. Repoli, M. J. Woodcock, H. I. M. Boshoff, O. Egbelowo, K. Gausi, P. Denti, F. Kaya, M. Zimmerman, E. L. Miller, V. A. Dartois, C. E. Barry, 3rd and B. B. Aldridge (2026). "PET/CT imaging of tuberculosis lung lesions in marmosets treated with different drug regimens aligns with human clinical outcomes." Sci Transl Med **18**(831): eado9383. DOI: 10.1126/scitranslmed.ado9383.

Sasahara, K., Y. Shimokawa, Y. Hirao, N. Koyama, K. Kitano, M. Shibata and K. Umehara (2015). "Pharmacokinetics and Metabolism of Delamanid, a Novel Anti-Tuberculosis Drug, in Animals and Humans: Importance of Albumin Metabolism In Vivo." Drug Metab Dispos **43**(8): 1267-1276.

Svensson, E. M., K. E. Dooley and M. O. Karlsson (2014). "Impact of lopinavir-ritonavir or nevirapine on bedaquiline exposures and potential implications for patients with tuberculosis-HIV coinfection." Antimicrob Agents Chemother **58**(11): 6406-6412.
Wang Q, Boshoff HIM. Determining Minimum Inhibitory Concentrations in Liquid Cultures or on Solid Medium. Methods Mol Biol. 2021;2314:595-609.

Wang Q, Boshoff HIM. Determining Minimum Inhibitory Concentrations in Liquid Cultures or on Solid Medium. Methods Mol Biol. 2021;2314:595-609. doi: 10.1007/978-1-0716-1460-0_26. PMID: 34235672; PMCID: PMC10500673.

White, A. G., P. Maiello, M. T. Coleman, J. A. Tomko, L. J. Frye, C. A. Scanga, P. L. Lin and J. L. Flynn (2017). "Analysis of 18FDG PET/CT Imaging as a Tool for Studying Mycobacterium tuberculosis Infection and Treatment in Non-human Primates." J Vis Exp(127).
